# Chikungunya outbreak in Bangladesh (2017): Clinical and hematological findings

**DOI:** 10.1371/journal.pntd.0007466

**Published:** 2020-02-24

**Authors:** Saeed Anwar, Jarin Taslem Mourosi, Md. Fahim Khan, Mohammad Ohid Ullah, Olivier M. Vanakker, Mohammad Jakir Hosen

**Affiliations:** 1 Department of Genetic Engineering and Biotechnology, School of Life Sciences, Shahjalal University of Science and Technology, Sylhet, Bangladesh; 2 Department of Statistics, School of Physical Sciences, Shahjalal University of Science and Technology, Sylhet, Bangladesh; 3 Center for Medical Genetics, Ghent University Hospital, Ghent, Belgium; Mahidol University, THAILAND

## Abstract

**Introduction:**

A massive outbreak of chikungunya virus (CHIKV) occurred in Bangladesh during the period of April-September 2017, and over two million people were at risk of getting infected by the virus. A prospective cohort of viremic patients was constituted and analyzed to define the clinical, hematological, and long-term aspects of this outbreak.

**Methods:**

A 35-day long comprehensive survey was conducted in two major, neighboring cities, Dhaka and Mymensingh. One-hundred and eighty-seven laboratory-confirmed CHIKV cases were enrolled in the cross-sectional cohort study. Additionally, a smaller group of 48 chikungunya patients was monitored for post-infection effects for 12 months.

**Results:**

Clinical data revealed that a combination of fever and arthralgia (oligoarthralgia and/or polyarthralgia) was the cardinal hallmark (97.9% of cases) of the infection. Hematological analysis showed that irrespective of age and sex groups, CHIKV patients had a decreased level of hemoglobin (n = 64, *p* < 0.01) and elevated erythrocyte sedimentation rate (n = 131, *p* < 0.01). Besides, a significant portion of the patients represented abnormal values for RBC (n = 38, *p* = 0.0005) and WBC (n = 63, *p* < 0.01) counts. The post-infection study revealed that children had an early recovery from the infection compared to the adults. Moreover, post-infection weakness, successive relapse of arthralgic pain, and memory problems were the most significant aftereffects, which had an impact on the daily activities of patients.

**Conclusions:**

This study represents a comprehensive overview of clinical and epidemiological features of the 2017 outbreak of CHIKV in Bangladesh as well as its chronic outcomes till the 12^th^ month. It provides insights into the natural history of this disease, which may help to improve the management of CHIKV patients.

## Author summary

The clinical profile, epidemiology, and the economic impacts during the acute phase of chikungunya infection have been studied quite rigorously. However, studies regarding the hematological features and chronic consequences are infrequent. In this study, we analyzed the clinical and hematological features of 187 chikungunya patients in the acute phase of the infection. Also, we monitored a smaller group of 48 patients until 12 months to study its post-infection consequences. Clinical data revealed that a combination of fever and joint pain (arthralgia) was the cardinal hallmark in the acute phase of the infection. Hematological analysis showed that CHIKV infection features a significantly reduced hemoglobin and remarkably elevated erythrocyte sedimentation rate. Besides, RBC and WBC counts, especially in children and females, were beyond the reference values. The post-infection consequence study unveiled that children recovered better from the infection compared to the adults. Further, post-infection weakness, successive relapse of joint pain and memory problems were the most significant aftereffects. Overall, the infection had a moderate to severe impact on the daily activities of the respondents. This study provides insights into the clinical and hematological aspects of chikungunya infection during the acute phase as well as describes an account for its chronic outcomes, which puts forward to the knowledge for clinicians and epidemiologists regarding the infection diversity and to help improve patient management.

## Introduction

Chikungunya is a neglected tropical disease, usually endemic to Africa, Southern and Southeast Asia. The causal agent of this disease is the chikungunya virus *(CHIKV)*, a classical arbovirus which possesses a single-stranded positive-sense RNA genome that is transmitted to humans through the bites of infected female Aedes mosquitoes, predominantly by *Aedes aegypti* and *Aedes albopictus* [1–4]. The general symptoms of CHIKV infections are frequently shared with a wide range of tropical infections, e.g., dengue, leptospirosis, rickettsioses, and are often tricky to differentiate clinically. Consequently, the actual burden of CHIKV zoonoses remains poorly understood and underestimated, and continually growing [5]. In 2013–14, World Health Organization reported the first local transmission of CHIKV in the Americas [6]. Epidemiologic and disease dynamics studies revealed that the virus infects around 3 million people each year, and a total of 1.3 to 2.7 billion people are currently living in areas at-risk of CHIKV transmission [6, 7].

Like many of the viral zoonoses in the tropics, CHIKV lacks any specific nor pathognomonic symptoms. In the most typical form of the infection (72–97% of cases), acute commencement of fever and polyarthralgia predominantly in the limb extremities is reported after a short incubation period of about 1 to 5 days [1, 3, 8]. Other symptoms include skin rash, headache, back pain, myalgia, and nausea [2, 9, 10]. Joint pain can often be severe and may remain indefatigable for weeks to years. The most severe form of the disease is often associated with neurological, cardiovascular, hepatic, dermatological, respiratory symptoms, along with miscarriages and neonatal infections [11–17]. Although only a few patients require hospitalization, there have been a few reports of fatalities due to CHIKV infection [18–21].

CHIKV can infect a significant proportion of a population within a short period, and it affects the productivity of infected individuals to a great extent. Hence, outbreaks of this virus do not only affect the infected individual or his/her family but the entire community. The debilitating disease poses a massive burden to the entire health infrastructure of a country, especially in low and lower-middle-income countries like Bangladesh. It is generally recommended that countries should develop and maintain the capacity to diagnose cases, manage patients, and employ social communication strategies to reduce the presence of the mosquito vectors. However, both capacity building and access to a healthcare facility are perhaps very challenging in countries like Bangladesh, and consequently, the actual prevalence, clinical extents, and overall burden are still elusive [20].

Till now, outbreaks of chikungunya have been reported in more than 60 countries [10]. The Indian subcontinent is one of the endemic areas of CHIKV. Since 2000, this subcontinent has experienced at least 11 major outbreaks of CHIKV, among which Bangladesh suffered a massive outbreak in 2017 and two comparatively smaller outbreaks in 2008 and 2011 [22–25]. The first recognized outbreak of chikungunya in Bangladesh came in 2008 from two villages of the northwestern region [23]. A detailed report on the etiology and clinical presentations of that outbreak is missing. Later, in November 2011, another chikungunya outbreak was reported in the Dohar-Dhaka area [24]. No fatal cases were reported; the clinical manifestations of this outbreak remained consistent with the classical forms of CHIKV infection, though the attack rate (~30%) within the circulation area was high [24, 26]. Although there is inconsistency regarding data on which age and gender group underwent most sufferings, children were less vulnerable and showed better recovery [21, 26]. Reports of sporadic cases of CHIKV infection continued to come in 2013, 2015, and 2016 [27]. The most dangerous outbreak of chikungunya in Bangladesh was reported in April—September 2017, when a massive number of positive cases were reported from 23 districts of the country; >13,000 clinically confirmed cases were documented in the city of Dhaka alone [25, 28–32]. The CHIKV from the 2017 outbreak in Dhaka was found to be genetically distinct from the strain found in the previous outbreak, Bangladesh/0810atw [33]. Phylogenetic analysis revealed that the outbreak strains constituted a new cluster within the Indian Ocean clade, suggesting that they are novel variants [34]. Together with variability in symptoms, 83% of patients in Dhaka also had ‘low’ to ‘very low’ overall quality of life, and ~30% of patients had ambulatory problems due to severe arthropathy [35]. However, the impact of CHIKV infection on hematological indices and its long-term effects have not yet been studied.

In this study, we assessed the complete blood counts in a cohort of 187 CHIKV patients enrolled during the CHIKV outbreak 2017 in Bangladesh. Besides, we have investigated the clinical features of these patients while a subgroup of this cohort was continuously followed until 12 months post-infection to understand the long-term effects better.

## Materials and methods

### Ethics statement

The methods and protocols used for this study were reviewed and endorsed by the Graduate Research Ethics Committee (Headed by the Dean), School of Life Sciences, Shahjalal University of Science and Technology. All participants or their legal representative gave written informed consent according to the Declaration of Helsinki.

### Patient recruitment and data collection

**Fig 1:**
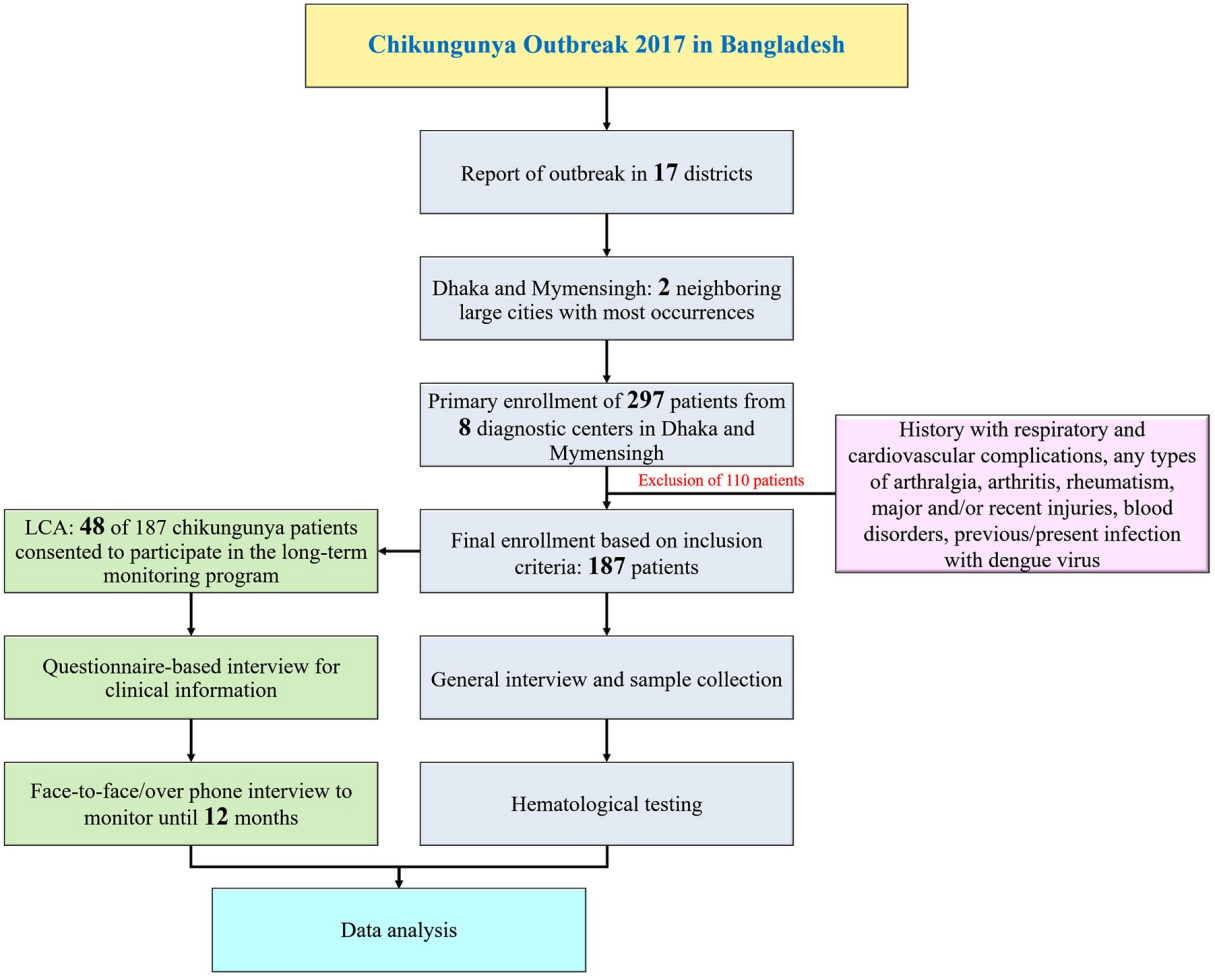
Study flow chart.

From June 30, 2017, to August 4, 2017, we recruited 297 laboratory-confirmed CHIKV cases of all age groups from Dhaka and Mymensingh districts (Fig 1). A laboratory-confirmed case was defined as a patient with detectable CHIKV RNA by RT-PCR or anti-CHIKV antibody by ELISA. A cross-sectional study was done to investigate the clinical, biochemical, and hematological profiling, and a long-term follow-up was conducted to understand the aftereffect of chikungunya on the quality of life. Primarily we recruited 297 laboratory-proven cases of CHIKV infection. Since Bangladesh lacks any functional referral system and a clinical record-keeping system like developed countries, we reached out to every patient to verify the diagnostic reports of CHIKV infection and collected scans or photocopies of the test reports as proof. However, patients (n = 99) with a history of respiratory and cardiovascular complications, previous reports of arthralgia, arthritis, rheumatism, any major recent injuries, or blood disorders were excluded from the study. Patients (n = 11) with proven evidence of previous or present infection by the Dengue virus were also not included in the study. So finally, this study was restricted in the analysis of clinical and hematological data of 187 patients. Among these 187 patients, 48 were found willing to follow a long-term monitoring scheme of 12 months (Long term consequence assessment group; LCA) (Fig 1). Of note, both the primary recruitment and final inclusion into the study were completely blinded from any age, sex, race, occupation, and economic condition related effects.

### Clinical, sero-biochemical and long-term effect study

Biochemical and serological test results were collected after they were prescribed and performed by a specialist physician and a specialist diagnostic center, respectively. Hematological reference ranges were defined as the set of values 95% of the healthy people fall within. We interviewed all the patients with a detailed questionnaire (S1 Fig). A team comprising of graduate students who majored in health and life sciences, clinicians, pediatricians, and statisticians was involved in administering the questionnaire-based survey. Two of our team members, who had experience in conducting questionnaires among children, specially interviewed the CHIKV positive children with easy and careful wording in the presence of his/her parents and caregivers.

The relative intensity of joint pain was evaluated using a numerical rating (NR) scale starting from 0 to 10. A rating of 0 indicated that the individual had no joint pain, and a rating of 10 indicates intolerable joint pain. Using these relative scores given by the patients, the pain intensity was categorized as mild (NR 1–4), moderate (NR 5–7), and severe (8–10). The anatomical location(s) of the pain and how long it existed after CHIKV infection were also documented.

After 2 months (**±** 3 days, M2) from day 0, all patients were asked for their condition. For long-term consequences assessment (LCA) of the after-effect of chikungunya, consented patients were interviewed with a standard questionnaire 4, 6, 9, and 12 months after the viremic phase (M4, M6, M9, and M12) (S1 Fig**)**. A team comprising of graduate students who majored in life sciences and public health disciplines administered the questionnaire.

Data were analyzed using SPSS (Statistical Package for Social Sciences) and statistical significance was tested using and two-tailed experiments including Student t-test, χ2 test, Fisher’s exact test, Spearman’s rho test (ρ_s_) test, and McNemar tests (for matched pairs of subjects) at a α-levels of 0.05 and 0.01 (*p* = 0.05 and *p* = 0.01, respectively)..

## Results

### Features of the patient’s cohort

**Fig 2:**
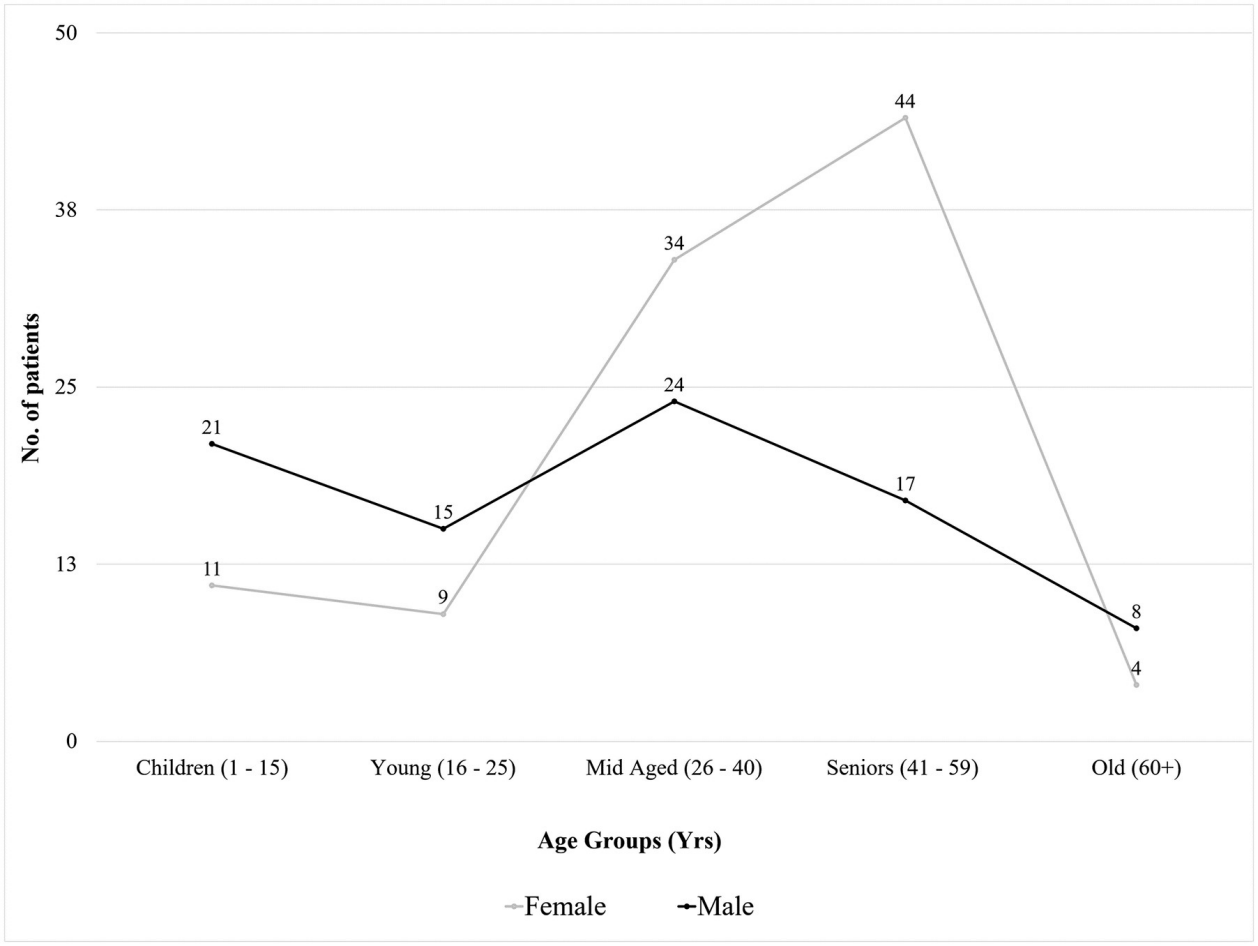
Age and gender distribution of the sample pool.

Among the 187 confirmed (using RT-PCR and/or immunological techniques) chikungunya (Table 1) patients, 117 (62.6%) patients were from the Dhaka region, while 70 (37.4%) were from Mymensingh. Interestingly, 18 patients from Mymensingh reported that they traveled to Dhaka in the weeks before the inclusion. The age range of CHIKV positive patients was between 3 and 84 years, with the majority of cases involving the age group 41–59 years (Fig 2). Also, 32 children (**≤15 yrs)** were included in this study.

**Table 1:** Diagnostic outcomes of sero-samples.

Parameter	Finally enrolled patients n (%)	LCA group n (%)
Total number of patients	187 (100%)	48 (100)
By Immunochromatography	135 (72.2%)	
By IgM ELISA	48 (25.7%)	
By both immunochromatography and IgM ELISA	4 (2.1%)	

LCA: Long-term consequence assessment; n: number of respondents

### Demographic Data

Randomly collected samples and demographic data analysis revealed that females were more prone to chikungunya (M:F = 1:1.34) (Table 2). Among 187 patients, two were admitted to a hospital, and most of the patients visited doctors and/or a diagnostic center within, on average, 5.1 days after the first symptoms.

**Table 2:** Demographic features of CHIKV positive patients.

Characteristics	Finally enrolled patients Value	LCA group Value
Male: Female (Ratio)	1:1.34	1:1.08
Age in years (female) (mean ± standard deviation, median, interquartile range)	38.5 ± 15.56, 40, 23	33.42 ± 8.52, 32, 9
Age in years (male) (mean ± standard deviation, median, interquartile range)	29 ± 17.58, 30, 27	33.44 ± 11.42, 31, 18
Time (days) from onset to diagnostic center visit (mean days ± standard deviation, median, interquartile range)	5.1 ± 2.92, 5, 2	4.8 ± 1.61, 5, 3
Hospitalization	2 (0.54%)	0 (0.0%)

LCA: Long-term consequence assessment

### Signs, symptoms and clinical features

The symptoms of CHIKV infected patients are presented in Table 3. The most common feature of the CHIKV infection was high fever (mean oral temperature: 39.878°C or 103.78 °F) and arthralgia, found to be present in 183 (~98%) of patients. As the first clinical symptom, ~63% (117 patients of 187) of the participants’ arthralgia prior to fever, while others had fever before arthralgia. The onset of arthralgic pain was more frequently reported between days 1 to 3 in the infected persons (64.5%, 118 patients of 183) (S2 Fig). Over 85% of the patients experienced severe pain with a median NRS score of 9 (S3 Fig). Almost 60% (76 patients of 129) of the patients who experienced myalgia, reported that onset of myalgia was accompanied by arthralgia (Table 3, S2 Fig). Manifestations of cutaneous symptoms, including rashes and itching, usually appeared after the onset of arthralgia and myalgia, predominantly between days 4 to 6 (S2 Fig). Other noticeable symptoms included swelling, stiffness, and redness of joints, itching, headache, cough, insomnia, fatigue and dizziness (Table 3).

**Table 3:** Signs and symptoms recorded from CHIKV positive patients during the acute phase.

Symptoms	Presence (n, %)	Presence for (days; median, Interquartile range)
Fever	183, 97.86^a^	5.5, 3
Joint and body ache related complaints		
Arthralgia	183, 97.86	17.5, 12
Oligoarthralgia	80, 43.71	
Polyarthralgia	103, 56.29	
Continuous pain	133, 72.67	
Swelling of joints	136, 72.72	
Stiffness of joints	97, 51.87	
Redness of joints	51, 27.27	3, 2
Symmetrical trend of pain	117, 62.56	
Myalgia	129, 68.98	8, 5
Pain-fever correlation	97, 51.87	
Other types of pain		
Headache	117, 62.56^b^	5, 3
Throat pain	35, 18.71	4, 2
Abdominal pain	9, 4.81	3, 2
Pain behind eye	18, 9.62	3.5, 2
Cutaneous symptoms		
Rash	148, 79.14	4.5, 3
Itching	132, 70.58	3, 2
Ocular issues		
Enophthalmos/eye irritation/uveitis	7, 3.74	4, 1
Redness of eye	121, 64.7	3, 3
Respiratory complaints		
Catarrh-cough	82, 43.85	
Other Respiratory symptoms^c^	20, 10.7	
Gastrointestinal complaints		
Dysentery-like symptoms	98, 52.4^d^	
Vomiting/vomiting tendency	59, 31.55	5, 4
Other gastrointestinal complaints^e^	20, 10.7	
Miscellaneous		
Sore in mouth/oral ulcer	55, 29.41	5, 3
Dizziness	109, 58.3	
Fatigue	101, 54	
Disturbance of sleep	121, 64.7	7.5
Hospitalization	2, 1.1	4.5, 1

^a^~2/3^rd^ reported continuous fever

^b^1/3^rd^ reported severe and continuous headache

^c^includes difficulties in breathing, short breathing and mucus

^d^half of the patients had dysentery-like symptoms during the whole course of disease

^e^includes diarrhea, nausea, and gastroenteritis

**Fig 3:**
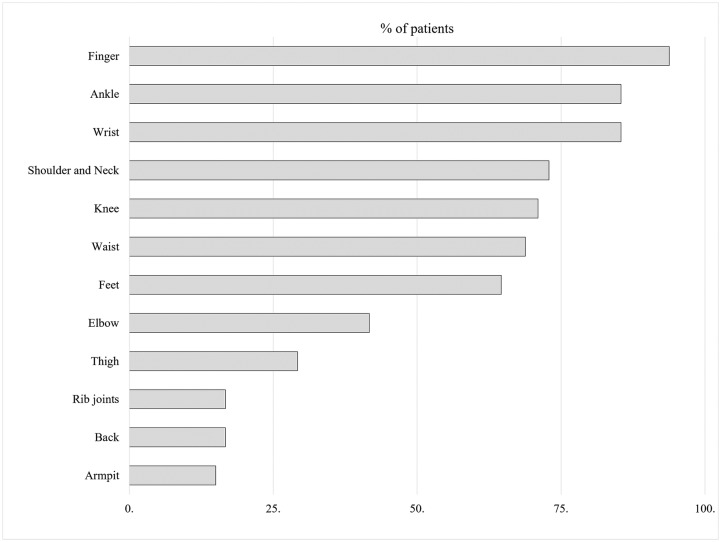
Sites of pain due to chikungunya infection.

Arthralgia was observed at 12 different anatomical sites (Fig 3), with hand joints (fingers and wrist), leg joints (ankle, knee, and feet), and shoulder and neck joints being most often affected in the chikungunya patients. Importantly, over 60% or our participants (n = 117, 62.56%) reported a symmetric trend of arthralgia. The intensity of the pain was stronger when patients tried to move. None of the patients reported arthralgia specific to a single anatomical site. Other signs and symptoms which were less frequent included gastrointestinal and respiratory complaints.

The signs and symptoms pattern of chikungunya seemed remarkably different in children compared to adults (Table 4). During the acute phase of the infection, arthralgia was less present (n = 28, 87.5%) while vomiting (n = 20, 62.5%) and headache (n = 20, 62.5%) was more frequently reported in children. In addition, the frequency of skin rash was notably higher (n = 136, 87.7%) in the adults.

**Table 4:** Differences in clinical manifestations of CHIKV positive children and adults.

	Children (≤15 yrs) (%)	Adults (>15 yrs) (%)	*p* value*
***Symptoms at onset (day 0 –day 7)***			
Arthralgia	28, 87.5	155, 100	0.0007
Headache	27, 84.37	89, 57.4	0.0046
Rash	12, 37.5	136, 87.7	< 0.01
Itching	14, 43.75	118, 76.1	0.0005
Vomiting	20, 62.5	39, 25.16	< 0.01
***Symptoms at M2***			
Arthralgia	5, 15.63	58, 37.41	0.0227
Headache	0, 00.0	13, 8.38	0.1295
Vomiting/vomiting tendency	0, 00.0	16, 10.32**	0.0784 0.0113***

*Calculated from Fisher’s exact test statistic

**All respondents were females

***Only female respondents were considered

### Hematological findings

**Fig 4:**
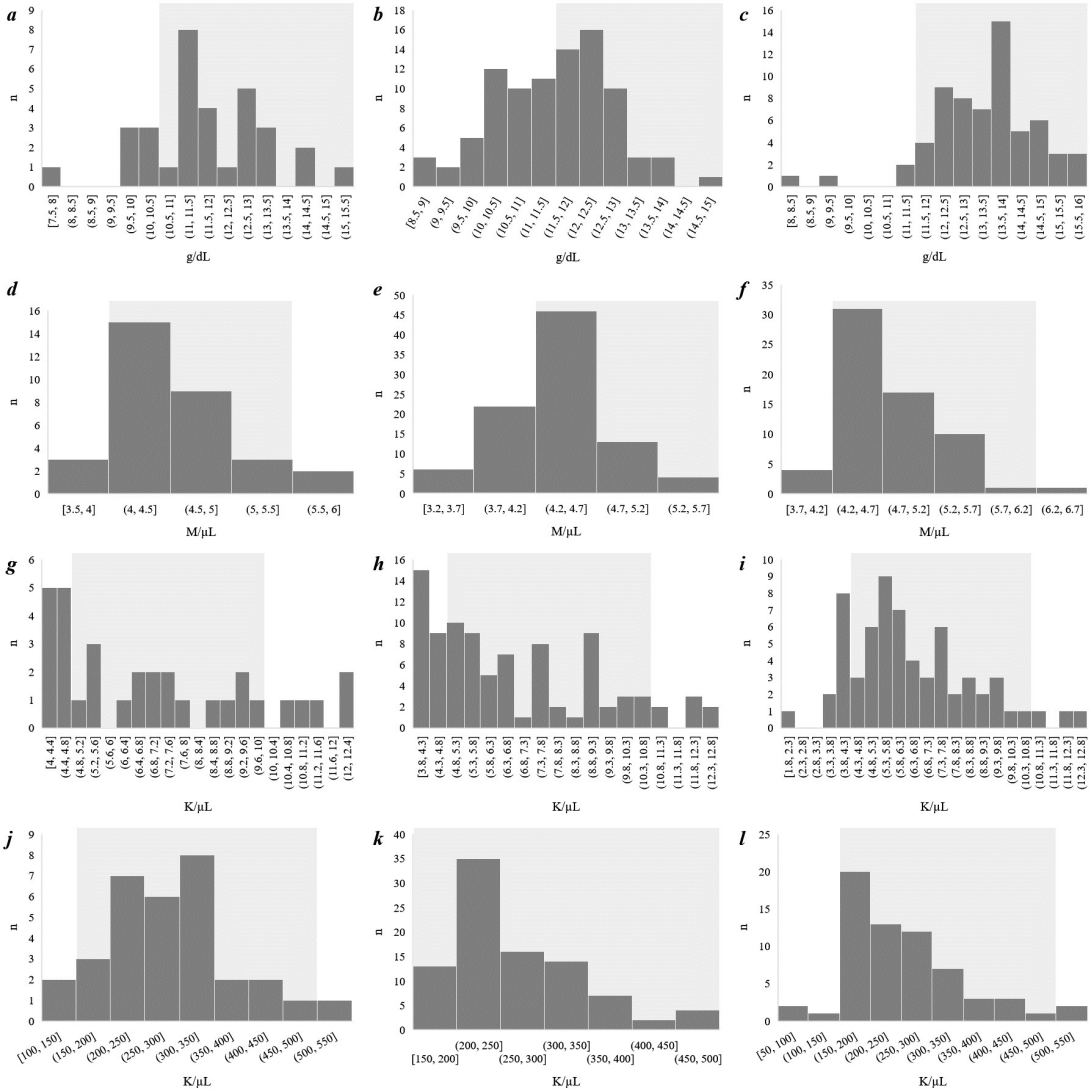
Histogram representing the major hematological findings from CHIKV positive patients. Shadowed area presents the reference ranges. a) Hemoglobin level in children. b) Hemoglobin levels in females. c) Hemoglobin level in males. d) RBC counts in children. e) RBC counts in females. f) RBC counts in males. g) WBC counts in children. h) WBC counts in females. i) WBC counts in males. j) Platelet counts in children. k) Platelet counts in females. l) Platelet counts in males.

Hematological analysis of CHIKV positive patients revealed that the hemoglobin level was significantly low in children (*p* = 0.0158) (Table 5). Among the adults <60 years of age, the median values for hemoglobin levels were within the recommended ranges; however, a significant portion of them (60 of 143 patients, 41.9%) had reduced counts (*p* <0.05). For CHIKV positive patients, the complete white blood cell (WBC) counts ranged from 2 to 12.6 K/μL, of which neutrophil (NTP) counts ranged between 32–80% and lymphocyte (LPC) counts ranged between 14–56%. Platelet counts ranged from 85 K/μL to 547 K/μL. Although the majority of the CHIKV positive patients were within normal ranges for whole WBC, neutrophils, LPC, and platelets (PLT), many patients represented varying degrees of lymphopenia when compared to reference values (Fig 4).

**Table 5:** Sex-specific hematological findings in CHIKV positive patients according to age groups.

	Median	Range, interquartile range	Within reference value n (%)	Beyond reference value n (%)	*p* value*
***Hemoglobin level in g/dL (Reference range for male*: *12–17 g/dL*, *female*: *11*.*5–15*.*5 g/dL*, *children*: *11–16 g/dL)***					
Children	11.55	7.82–15.2, 1.1	12 (37.5)	20 (62.5)	<0.01
Female	11.8	8.8–15, 1.75	53(58.24)	38 (41.76)	<0.01
Male	13.55	8.1–15.6, 1.9	58 (90.63)	6 (9.37)	0.1128
**Overall**	**12.1**	**7.82–16.5**	**123 (65.78)**	**64 (34.22)**	**<0.01**
***ESR in mm in 1st hour (Reference range for male*: *0–10 mm*, *female*: *0–20 mm*, *children*: *0–10 mm)***					
Children	25	4–164, 26.5	8 (25)	24 (75)	< 0.01
Female	31	7–111, 29	26 (28.57)	65 (71.42)	< 0.01
Male	15.5	3–104, 14	22 (34.37)	42 (65.63)	< 0.01
**Overall**	**25**	**5–164, 29**	**56 (29.95)**	**131 (70.05)**	**< 0.01**
***RBC count in M/μL (Reference range for adult*: *4*.*2–6*.*2 M/μL*, *children 4*.*0–5*.*5 M/μL)***					
Children	4.4	3.7–5.66, 0.7	27 (84.38)	5 (15.62)	0.0480
Female	4.3	3.2–5.7, 0.675	63 (69.23)	28 (30.77)	< 0.01
Male	4.7	3.7–6.4, 0.82	59 (92.18)	5 (7.12)	0.4627
**Overall**	**4.5**	**3.2–6.4, 0.7**	**149 (79.67)**	**38 (20.33)**	**0.0005**
***WBC count in K/μL (Reference range for adult*: *4*.*8–10*.*8 K/μL*, *children 4*.*8–10 K/μL)***					
Children	6.725	4–12.2, 7.3	17 (53.12)	15 (46.88)	< 0.01
Female	6	3–12.6, 4.3	60 (65.93)	31 (34.07)	< 0.01
Male	6	2–12.4, 3.65	47 (73.43)	17 (26.57)	< 0.01
**Overall**	**6**	**2–12.6, 4.2**	**124 (66.31)**	**63 (33.69)**	**< 0.01**
***Neutrophil part in % (Reference range*: *40–70%)***					
Children	57.5	37–72, 16.5	29 (90.63)	3 (9.37)	0.3666
Female	47	32–77, 12	81 (89.01)	10 (10.98)	0.1243
Male	63	46–80, 13	49 (76.56)	15 (23.44)	0.0004
**Overall**	**62**	**32–80, 14**	**159 (85.03)**	**28 (14.97)**	**< 0.01**
***Lymphocyte part in % (Reference range*: *20–45%)***					
Children	35	23–55, 8	29 (90.63)	3 (9.37)	0.3666
Female	32	15–65, 12	83 (91.2)	8 (8.8)	0.2987
Male	28	14–48, 13.5	54 (84.38)	10 (15.62)	0.0213
**Overall**	**32**	**15–65, 13**	**166 (88.77)**	**21 (11.22)**	**0.0797**
***Platelets count in K/μL (Reference range for adult*: *150–500 K/μL*, *children <10 yrs*: *150–550 K/μL*, *children >10 yrs*: *150–550 K/μL)***					
Children	263.5	115–505, 104.5	29 (90.63)	3 (9.37)	0.3666
Female	248	154–500, 101.5	91 (100)	0 (0.0)	-
Male	237	85–547, 112	59 (92.18)	5 (7.82)	0.4627
**Overall**	**250**	**85–547, 107**	**179 (95.72)**	**8 (4.28)**	**0.7793**

*calculated from χ2 test

Hematological analysis of CHIKV positive patients revealed that a significant portion of the patients (n = 64, *p* < 0.01), especially the children and women, had reduced levels of hemoglobin (Table 5). Our analysis showed that the hemoglobin level was significantly low in children (*p* = 0.0158) (S2 Table). Among the adults <60 years of age, the median values for hemoglobin levels were within the recommended ranges; however, a significant portion of them (n = 60, *p* <0.05) had reduced counts (S2 Table). Except for the platelets, a significant portion of the patients represented abnormal values for counts of RBC (n = 38, *p* = 0.0005) WBC (whole; n = 63, *p* < 0.01)) and parts of neutrophils (n = 28, *p* < 0.01) (Table 5). Regarding RBC counts, almost 1 in every 3 women (28 or 91 women, *p* < 0.01) showed reduced counts (Table 5). Across age and sex barriers, ~70% of patients showed moderate to highly elevated ESR (Table 5, S2 Table, Fig 4).

For all CHIKV positive patients, the complete white blood cell (WBC) counts ranged from 2 to 12.6 K/μL, of which neutrophil (NTP) counts ranged between 32–80% and lymphocyte (LPC) counts ranged between 14–56%. Platelet counts ranged from 85 K/μL to 547 K/μL. Although the majority of the CHIKV positive patients were within normal ranges for whole WBC, neutrophils, LPC, and platelets (PLT), many patients represented varying degrees of lymphopenia when compared to reference values (Table 5, Fig 4, S2 Table).

However, during the acute phase of the disease, no significant correlation was observed between the level of leukocytopenia and the intensity of arthralgic pain (n = 48, ρ_s_ = 0.02126, *p* = 0.88594, S3 Fig). Red blood cell (RBC) count was remarkably beyond the reference range in the mid-aged (n = 47, 81.03%) and senior groups (n = 44, 72.13%) (S2 Table). The interquartile range of the RBC was between 3.2 to 6.4 M/μL, and the median value was 4.5 M/μL. Erythrocyte sedimentation rate (ESR) was significantly higher in all age groups, especially in female patients (Table 5). Further, a significant correlation was obtained between the different age groups and RBC counts, neutrophil counts, and leucocyte counts of the CHIKV positive patients (S1 Table).

### Characteristics of long-term arthralgia in CHIKV infected patients

#### Long-term arthralgia associated with CHIKV infection

**Fig 5:**
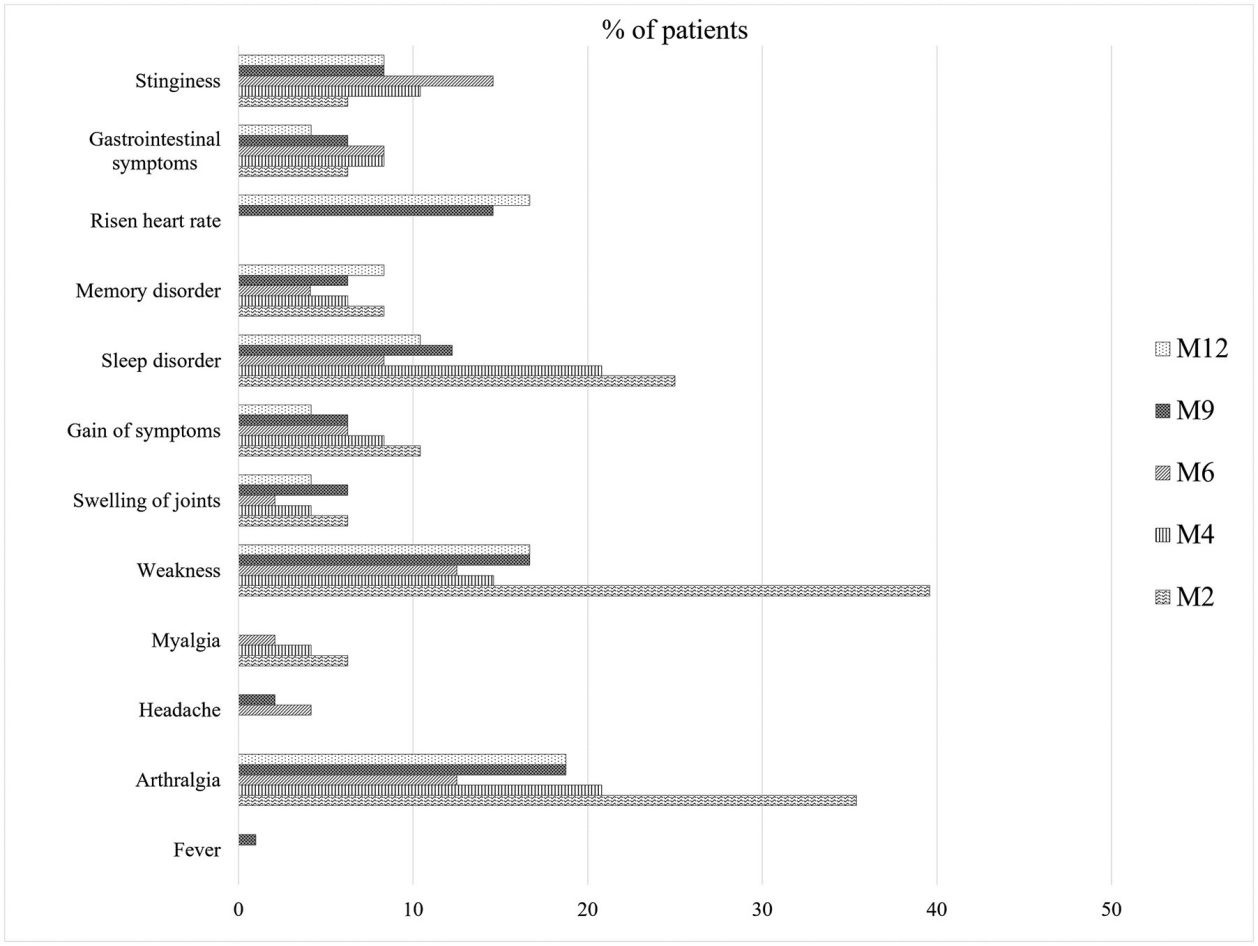
Persistence of chikungunya symptoms over time course in CHIKV positive patients.

All patients enrolled in the LCA group were interviewed using a questionnaire at M2, M4, M6, M9, and M12 post-CHIKV infection to monitor the persistence of fever, arthralgia, and other clinical symptoms. None of our monitored patients reported any relapse of the fever after M4, and episodic arthralgia was the most significant post-infection burden (S3A Table). ~23% of the patients (11 of 48) reported fever at either M2 or M4 with a duration of 1 to 2 days (median: 1 day, mean: 1.36 days) **(**S3A Table**)**; however, all of them commented that the intensity of the arthralgia and fever was not as high as they felt during the acute phase. The percentage of patients suffering from long-term arthralgia decreased significantly (till M6) after the acute phase of the infection and then raised to ~19% at M9 and M12. Most of our enrollees complained of intermittent arthralgia, with successive recovery and relapse; none of the patients complained of permanent arthralgia at any timepoint after the acute phase, and all of the respondents reported that the intensity of the pain was significantly reduced after M2. Of note, all of our enrolled patients in the LCA group suffered from arthralgia between days 0 to 7, and none of them suffered from joint pains before the CHIKV infection. The McNemar test for matched pairs of subjects revealed that the site of arthralgic pain (S3B Table) remained the same at each time point. The percentage of patients suffering from myalgia decreased a lot after the acute phase of the infection and stabilized by M6 (*p* <0.01). None of the patients reported having myalgia at M9 and M12 (Fig 5). When CHIKV-induced arthralgia relapsed, it was symmetrical, involving more than two different anatomical locations. Regardless of age and sex, finger joints (48 of 48, 100%), wrists (44 of 48, 91.67%), and ankles (44 of 48, 91.67%) were affected most frequently (S3B Table).

We noted that the number of sites affected by arthralgia gradually diminished in patients still suffering until M2, with only 23% of patients (n = 11) suffering from polyarthralgia. However, the number of anatomical locations further decreased significantly in M4 and M6 and then stabilized at M9 and M12 (50% and 64% respectively, *p* < 0.0001).

#### Other long-term clinical signs associated with CHIKV infection

At M6, M9, and M12, the LCA group displayed other symptoms, including local swelling of joints, cutaneous and dermatological symptoms, and post-infection weakness. Additionally, sleep, memory and/or concentration disorders, as well as depression and stinginess, were remarkably associated (Fig 5). Furthermore, between M6 and M12, 16.67% (n = 8) complained that they frequently suffered tachycardia during working, even though none of them had any previous heart complications.

After the acute phase of the infection, none of our patients reported visiting a physician until M4. However, the number of patients who visited a physician increased significantly between M6 (4; 8.33%) and M12 (16; 33.33%, S3C Table) (*p* < 0.01). The most significant after-effect of the infection in our study population appeared to be the post-infection weakness. Around 40% of the patients reported having a continuous weakness at M2. The percentage decreased significantly at M6, increasing however again to ~17% at M9 and M12. Besides, more than 20% of patients complained about sleep disorder at M2 and M4, but the percentage diminished to less than 10% at M6. However, complaints of disturbed sleep slightly increased at M9 and M12 (Fig 5).

Over 10% of patients complained of new symptoms at M2, which they had not suffered from during the acute phase of the infection (Fig 5). Even at M6, 6.25% of patients reported suffering new symptoms, and more than 4% of patients to experience new symptoms at M9. However, we did not attempt to find any association between the newly gained symptoms and the effect of the RNA virus infection. Although the new symptoms were generally sleeping problems, swelling of joints, arthritis-like symptoms, and memory problems were also reported. Although the respondents did not display any neurological dysfunction, the mild memory problems could not be excluded to result from the CHIKV infection. Some of the patients reported that they were frequently prone to depression and partially lost control over their temper.

### Post-infection impact on daily life at M6 and M12

Arthralgia coupled with weakness in patients at M6 and M12 were highly incapacitating for daily life activities, professional life, and leisure activities (Table 6). Most of the patients having chronic arthralgia complained of pain when rising from sitting and lying, walking, or picking up a load. ~27% (n = 13) and ~23% (n = 11) of patients respectively in M6 and M12 reported that arthralgia affected their professional activities. Remarkably, ~31% of patients (n = 15) complained that arthralgia had disturbed them in leisure. Moreover, the patients having memory problems (Fig 5) at different time points complained that it had a significant impact on their day to day life activities.

**Table 6:** Impact of arthralgia on daily life for patients at M6 and M12.

	M6 (%)	M12 (%)
**Issues regarding the quality of life**		
Discomfort while rising from sitting/lying	22.91	16.67
Weakness in long walks (over 1 KM)	10.41	8.33
Discomfort while picking up a heavy object	20.83	18.75
At least one of these discomforts	29.17	25
**Impacts on working life**		
With activity	27.1	22.91
Physical impact	22.91	10.41
low impact	54.54	55.55
moderate impact	27.27	22.22
high impact	18.18	22.22
**Impact on leisure-time**		
No impact	66.67	62.5
Physical impact	33.33	29.17
low impact	18.75	35.71
moderate impact	31.25	28.57
high impact	50	35.71

M: month

## Discussion

The chikungunya outbreak of 2017 in Bangladesh appeared as an epidemic manifestation, with 23 of 65 districts of the country infected. This study presents the clinical and epidemiological data of this chikungunya outbreak.

Bangladesh is a riverine monsoon country, and as such, an ideal vicinity for the emergence of arboviral diseases, including dengue and chikungunya. As both have overlapping pathophysiological mechanisms and proceed simultaneously, it is a real challenge for physicians to distinguish among them, especially during the early stages of infection [35].

CHIKV was found to infect all ages and both sexes; however, ratios varied. A higher percentage of cases was observed in adult females (56.7%) than males (38%) and female children (43.3%). However, previously published reports have indicated that both sexes suffer equal burdens of the disease [36, 37]. The higher percentage of adult female cases may be due to higher levels of exposure to infected vectors in the home environment since Bengali women spend more time at home, and the mosquitoes are commonly found indoors [38–41]. The difference in the number of cases in the age groups may not reflect the vulnerability of any specific groups but indicate the general population structure in the country [42], i.e., the infection trend was not biased to any age group.

Irrespective of sex, the combination of fever and severe arthralgia (present in 97.9% of cases) can be regarded as the cardinal hallmark of the chikungunya 2017 outbreak in Bangladesh. This is consistent with the previous outbreak report (83.3 to 98%), though the values were less in case of the children [43–46] (Tables 2 and 3). However, in an Indian outbreak of the virus in Kerala in 2007, arthralgia was found to be the initial symptom in only ~17% patients [47].h.

We found a symmetrical presentation of arthralgia in most of the cases (62.5%), while a higher percentage of patients reported polyarthralgia (56.25%) than oligoarthralgia (43.75%). Also, we observed that finger joints (93.8%) and wrist (85.4%) joints were the most affected sites. In the acute phase, the frequency of incapacitating pain involving certain peripheral joints (Fig 3) was found to be comparable with the study of Queyriaux et al. 2008 and Staikowsky et al. 2008; however, it contrasted with earlier reports from India and Suriname [47–51].

Other symptoms including headache, itching, catarrh-cough, dizziness, and dysentery-like symptoms (passage of blood through the anus, with stools) were found to be similar to most of the previous studies, except for an unusually high frequency of rash (79.2%), swollen joints (72.9%) and redness of the eyes (64.17%) in the present study [52].

Over 85% (41 of 48) of the patients complained of severe pain with a median NRS score of 9 throughout the acute phase (S3 Fig), which was similar to the findings of Staikowsky et al. 2008 [49]. Almost two-thirds (64.6%) of our enrolled patients faced sleep disturbances due to arthralgia and myalgia (Table 3). The rate of hospitalization (1.1%) was very low, and the outbreak did not cause any fatal outcomes. Other studies on different cohorts but the same outbreak also represented low hospital admission rates; however, exclusion of comorbid cases could be a reason behind the even lower hospitalization in this study [35, 53]. To mention, there is yet to find a report on CHIKV-associated mortality from this outbreak. From the overall severity and the extent of arthralgia-related manifestations, it may be perceived that an aggressive strain of CHIKV probably circulated during the outbreak [35]. However, molecular characterization of the CHIKV isolates collected during this outbreak that these strains belonged to the Indian Ocean clade of the East/Central/South African (ECSA) genotype and were lacking the aggressive Ala226Val substitution [34]. It suggests that the strains circulated during the outbreak caused non-fatal and non-severe infections only. It is important to keep in mind that the data analyzed in this study included, perhaps, only the non-severe cases.

The severity of certain clinical manifestations of chikungunya may perhaps depend on several factors, including age, gender, immune status, genetic predisposition, etc. [52]. Our analysis showed that children (<15 years) had a lower tendency to have skin rash and itching as well as vomiting. Conversely, a significantly higher frequency of headache was observed among the children compared to other age groups. The duration of pain and rate of any relapse of post-infection symptoms (until M2) were significantly lower among children as compared to other age groups (Table 4). Interestingly, 16 females but no children or male patients reported occasional vomiting tendency at M2 (Table 4).

With regard to the hematological indices, distinct CHIKV markers are yet to be found. Lee et al. (2012) documented several predictable laboratory tests for detecting CHIKV, e.g., a drop-in lymphocyte count and a higher count of platelets, leukocytes, and neutrophils [54]. In our study, significant differences from hematological reference ranges were documented in all age and sex groups (Table 5, S2 Table). The hemoglobin level was significantly lower in children and women; however, RBC counts were significantly beyond the reference range in mid-aged and senior groups. Almost three of every four (28 of 38) and three of every five (38 of 64) patients who had reduced RBC and hemoglobin counts, respectively, were female. We were not able to document any significant drop in the lymphocyte parts on WBC nor any considerable increase in platelets (Table 5). These outcomes are atypical when compared to the reports from the Ahmedabad outbreak, the Caribbean outbreak in Trinidad in 2015, the La Romana outbreak in 2016, and the Kandy outbreak in Sri Lanka in 2006–07 [55, 45, 56–57]. However, the ESR values obtained in our analysis reports a broad range with significantly elevated rates in most cases across age and sex groups (*p* < 0.01) (Table 5, S2 Table). Based on the studies on blood, we do not claim that the hematological differences are solely due to CHIKV infections; however, this study sheds light on the fact that the viral infection could be a significant contributor behind these abnormalities. Besides, the socio-demographic background of the patients could have an effect on the outcomes. Nonetheless, these outcomes shed light on the fact that CHIKV associated blood indices require to be studied more extensively.

Based on the follow-up of patients with acute CHIKV infection who consented to participate, this study shows the evolution of arthralgia, mapping the frequency and location of arthralgic sites during a 12-month time period. Our data reveals that the proportion of patients having CHIKV-induced arthralgia decreased at an almost constant rate at each time point (Fig 5). Myalgia was not a complaint anymore at M6 and thereafter. This is different compared to the higher percentage of patients with long-term symptoms was reported by several studies of Italian and French cohorts of La Re´union Island or metropolitan France [44, 58–60]. Till M12, CHIKV-induced arthralgia was mainly symmetrical, and finger joints, wrist, ankle, and knee were found to be most affected; this remains consistent with other studies [44, 56].

There is evidence from different countries—notably France, India, Sri Lanka, Malaysia, Colombia, Venezuela, and the USA—to suggest that a sudden rise of heart rate was associated with the infection at both the acute and the chronic phases [61]. In our cohort, cardiovascular manifestations were not reported by patients during the acute phase and till M6. However, 16.67% of the patients experienced abnormal heart rates between M6 and M12. Alvarez et al. underlined the urgent need to explore the cardiovascular impact of a CHIKV infection in 2017 [61]. To date, these effects remain to be elucidated.

Weakness during professional activities was noted to be the most prominent after effect of the infection, as almost 40% of our patients reported to have severe weaknesses at M4. The proportion diminished over time but relapsed several times in some patients till M12. In addition, many patients complained of disturbed sleep, swelling of joints, and suffered new symptoms, e.g., memory problems. Although the patients in our study did not display any significant neurological symptoms at the acute phase of disease, we were unable to exclude that these memory problems during the chronic phase resulting from CHIKV spread in the central nervous system, as it had been reported that CHIKV disseminates to the central nervous system in humans and animals [62–65]. As was evident in other studies, chronic CHIKV induced complications are considered incapacitating for daily life tasks and impact professional activities and quality of life [59–60].

While the previous studies concerning chikungunya outbreak 2017 in Bangladesh were limited within the samples recruited from Dhaka only, this study represents a diverse sample population [35, 53, 66]. In addition, our study was extended to the hematological and chronic outcomes of the outbreak rather than to be confined only within the study of clinical and quality of life parameters [35]. However, this study is not free from any limitations. This recruited only the laboratory-confirmed cases of chikungunya, but the studied sample pool was relatively smaller than the previous study [35, 66]. Hossain et al., 2018 reported that the representation of laboratory-confirmed cases of CHIKV was very low during the 2017 outbreak in Bangladesh due to the high cost of testing and scarcity of diagnostic facilities [35]. Actually, during the pick of the outbreak, the Directorate General of Health Services (DGHS, the health service regulatory authority of Bangladesh) asked suspected patients not to seek laboratory tests [67]. DGHS was driven to circulate this instruction based on two principal reasons–(1) the average income of a significant portion of the people at risk was low and (2) the case fatality rate of chikungunya is generally insignificant [35, 67]. Since we enrolled only laboratory-confirmed cases of CHIKV infection in this study, this might be an explanation behind the recruitment of only a moderate-sized sample. However, after the outbreak was stabilized, there was no instruction from the DGHS about the possible chronic effects of the infection. So, when a significant portion of the patients who had the infection continued to face troubles in their day-to-day lives, it started to grow concern among them again, and many of them visited a physician during this stage. Broadly, it highlights the inefficiency of DGHS in health service delivery in Bangladesh.

Moreover, data regarding the clinical, chronic impact, and daily-life related parameters were collected through retrospective technique, which might be prone to the incompleteness of recalling. It is not unlikely that some respondents have overvalued some clinical symptoms due to the psychological impacts of massive social media coverage of the outbreak. Nevertheless, this study was conducted during the very peak of the outbreak, and the patients were monitored and interviewed rigorously at regular intervals; we assume any potential bias due to the incompleteness of recalling was minimized. Besides, this study involved the participation of the children, and a child may indeed respond differently as compared to an adult. Both children and older individuals may be prone to response effects, particularly when the question demands information retrieved from memory. Hence, the data presented on the effects of CHIKV infection on children may contain biases due to response effects; however, we tried to reduce the impacts of these types of biases.

In summary, this study alludes to the clinical and epidemiological characteristics of the chikungunya outbreak of 2017 in Bangladesh. It facilitates our comprehension of the pathophysiology of the disease across age and sex groups and its chronic consequences till M12, a prerequisite for the development of efficient management and therapeutic strategies and for assessing the damage inflicted upon the population by a chikungunya outbreak.

## Supporting information

S1 Checklist STROBE Checklist. (DOCX)

S1 Fig Questionnaire form used for acquiesced medical data collection during and after the chikungunya outbreak 2017 in Bangladesh. (DOCX)

S2 Fig The onset of major symptoms during the acute phase of CHIKV infection. (DOCX)

S3 Fig The intensity of arthralgia based on NRS. (DOCX)

S1 Table Correlation between age and hematological data. (DOCX)

S2 Table Age stratified hematological findings in CHIKV positive patients. (DOCX)

S3 Table A: LCA study. Demographic information of the patients as well as the presence of fever and joint pain at different time points. B: Outcomes of McNemar test. C: Number of patients visited a physician due to post-CHIKV complications (n = 48).
(DOCX)
