# Supplementary material for: Chikungunya outbreak in Bangladesh (2017): Clinical and hematological findings
Source: PLoS Negl Trop Dis. 2020 Feb 24;14(2):e0007466. doi: 10.1371/journal.pntd.0007466 (PMC7058364; doi:10.1371/journal.pntd.0007466)
Supplement: S1 Checklist — (DOCX) [file pntd.0007466.s001.docx]

**STROBE Statement**

Checklist of items that should be included in reports of ***cohort studies***

|  | Item No | Recommendation | Where found in the manuscript | |
| --- | --- | --- | --- | --- |
| **Title and abstract** | 1 | (*a*) Indicate the study’s design with a commonly used term in the title or the abstract | In the abstract, paragraph 2 | |
|  |  | (*b*) Provide in the abstract an informative and balanced summary of what was done and what was found | In the abstract. | |
| Introduction | | |  | |
| Background/rationale | 2 | Explain the scientific background and rationale for the investigation being reported | Abstract, paragraph 1  Introduction, paragraphs 1, 2 | |
| Objectives | 3 | State specific objectives, including any prespecified hypotheses | Introduction, paragraph 2. | |
| Methods | | |  | |
| Study design | 4 | Present key elements of study design early in the paper | Methods, paragraph 1, 3 | |
| Setting | 5 | Describe the setting, locations, and relevant dates, including periods of recruitment, exposure, follow-up, and data collection | Methods, paragraph 1 | |
| Participants | 6 | (*a*) *Cohort study*—Give the eligibility criteria, and the sources and methods of selection of participants. Describe methods of follow-up  *Case-control study*—Give the eligibility criteria, and the sources and methods of case ascertainment and control selection. Give the rationale for the choice of cases and controls  *Cross-sectional study*—Give the eligibility criteria, and the sources and methods of selection of participants | Methods, paragraph 1 | |
|  |  | (*b*) *Cohort study*—For matched studies, give matching criteria and number of exposed and unexposed  *Case-control study*—For matched studies, give matching criteria and the number of controls per case |  | |
| Variables | 7 | Clearly define all outcomes, exposures, predictors, potential confounders, and effect modifiers. Give diagnostic criteria, if applicable | Methods, paragraph 1, 3 | |
| Data sources/ measurement | 8* | For each variable of interest, give sources of data and details of methods of assessment (measurement). Describe comparability of assessment methods if there is more than one group | Methods, paragraph 1 | |
| Bias | 9 | Describe any efforts to address potential sources of bias | Discussion, paragraph 13 | |
| Study size | 10 | Explain how the study size was arrived at | Discussion, paragraph 13 | |
| Quantitative variables | 11 | Explain how quantitative variables were handled in the analyses. If applicable, describe which groupings were chosen and why | Methods, paragraphs 1, 3. | |
| Statistical methods | 12 | (*a*) Describe all statistical methods, including those used to control for confounding | Methods, paragraph 3 | |
|  |  | (*b*) Describe any methods used to examine subgroups and interactions | Methods, paragraph 3 | |
|  |  | (*c*) Explain how missing data were addressed |  | |
|  |  | (*d*) *Cohort study*—If applicable, explain how loss to follow-up was addressed  *Case-control study*—If applicable, explain how matching of cases and controls was addressed  *Cross-sectional study*—If applicable, describe analytical methods taking account of sampling strategy |  | |
|  |  | (*e*) Describe any sensitivity analyses |  | |
| Results | | | |  |
| Participants | 13* | (a) Report numbers of individuals at each stage of study—eg numbers potentially eligible, examined for eligibility, confirmed eligible, included in the study, completing follow-up, and analysed | |  |
|  |  | (b) Give reasons for non-participation at each stage | |  |
|  |  | (c) Consider use of a flow diagram | | Methods, paragraph 1 (Fig 1) |
| Descriptive data | 14* | (a) Give characteristics of study participants (eg demographic, clinical, social) and information on exposures and potential confounders | | Table 1, 2, Fig 1 |
|  |  | (b) Indicate number of participants with missing data for each variable of interest | |  |
|  |  | (c) *Cohort study*—Summarise follow-up time (eg, average and total amount) | | Results, paragraph 7, 8, 9 Fig 5, S4 Table |
| Outcome data | 15* | *Cohort study*—Report numbers of outcome events or summary measures over time | | Results, paragraph 7, 8, 9 Fig 5, S4 Table |
|  |  | *Case-control study—*Report numbers in each exposure category, or summary measures of exposure | |  |
|  |  | *Cross-sectional study—*Report numbers of outcome events or summary measures | |  |
| Main results | 16 | (*a*) Give unadjusted estimates and, if applicable, confounder-adjusted estimates and their precision (eg, 95% confidence interval). Make clear which confounders were adjusted for and why they were included | | Table 2, 3, 4 |
|  |  | (*b*) Report category boundaries when continuous variables were categorized | |  |
|  |  | (*c*) If relevant, consider translating estimates of relative risk into absolute risk for a meaningful time period | |  |
| Other analyses | 17 | Report other analyses done—eg analyses of subgroups and interactions, and sensitivity analyses | | S4 Table |
| Discussion | | | |  |
| Key results | 18 | Summarise key results with reference to study objectives | | Discussion, paragraphs 3, 4, 5, 7, 9, 11 |
| Limitations | 19 | Discuss limitations of the study, taking into account sources of potential bias or imprecision. Discuss both direction and magnitude of any potential bias | | Discussion, paragraph 13 |
| Interpretation | 20 | Give a cautious overall interpretation of results considering objectives, limitations, multiplicity of analyses, results from similar studies, and other relevant evidence | | Discussion, paragraph 14 |
| Generalisability | 21 | Discuss the generalisability (external validity) of the study results | | Discussion, paragraph 14 |
| Other information | | | |  |
| Funding | 22 | Give the source of funding and the role of the funders for the present study and, if applicable, for the original study on which the present article is based | |  |

*Give information separately for cases and controls in case-control studies and, if applicable, for exposed and unexposed groups in cohort and cross-sectional studies.
