# Supplementary material for: Chikungunya outbreak in Bangladesh (2017): Clinical and hematological findings
Source: PLoS Negl Trop Dis. 2020 Feb 24;14(2):e0007466. doi: 10.1371/journal.pntd.0007466 (PMC7058364; doi:10.1371/journal.pntd.0007466)

Questionnaire form used for acquiesced medical data collection during and after the chikungunya outbreak 2017 in Bangladesh.


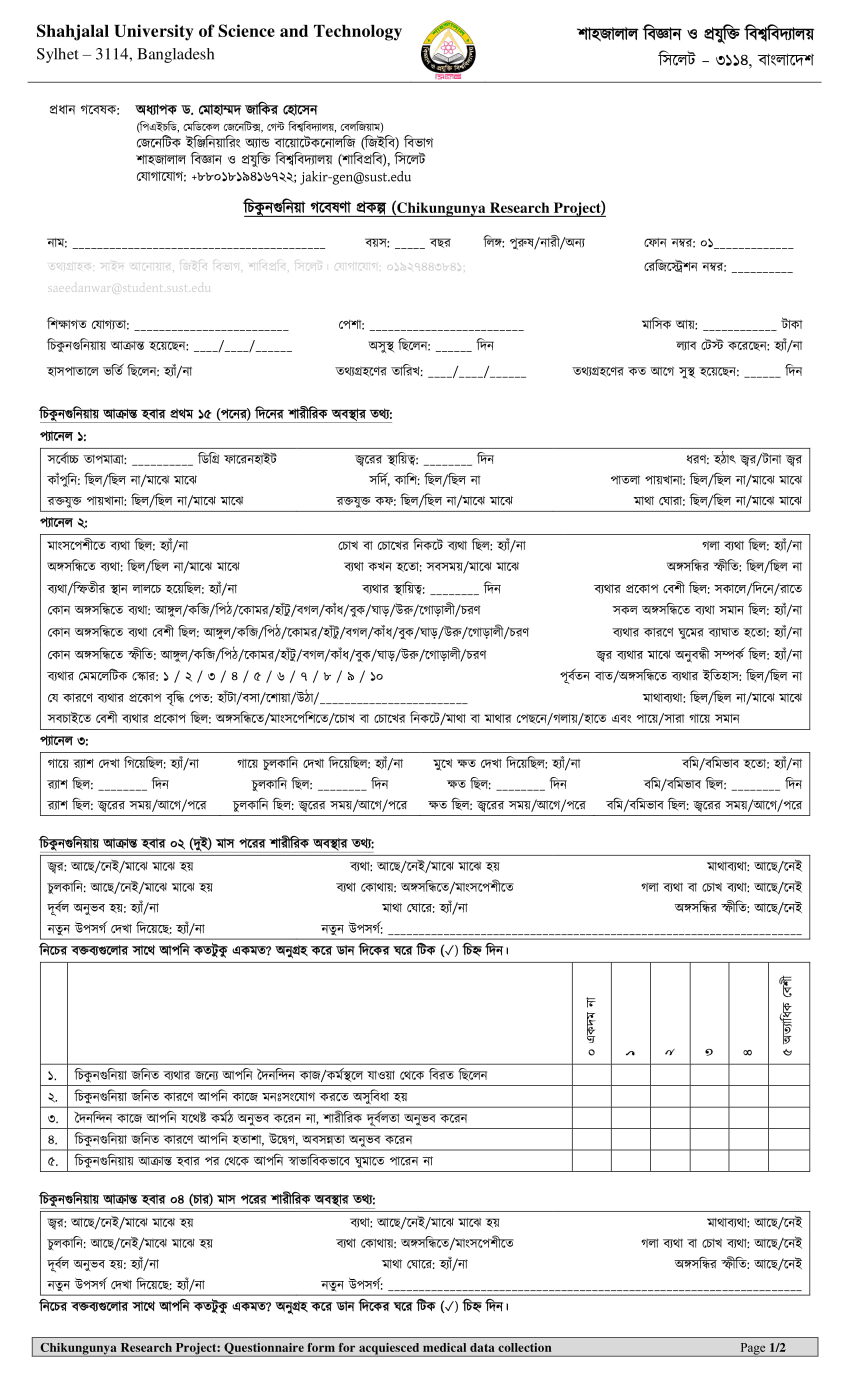


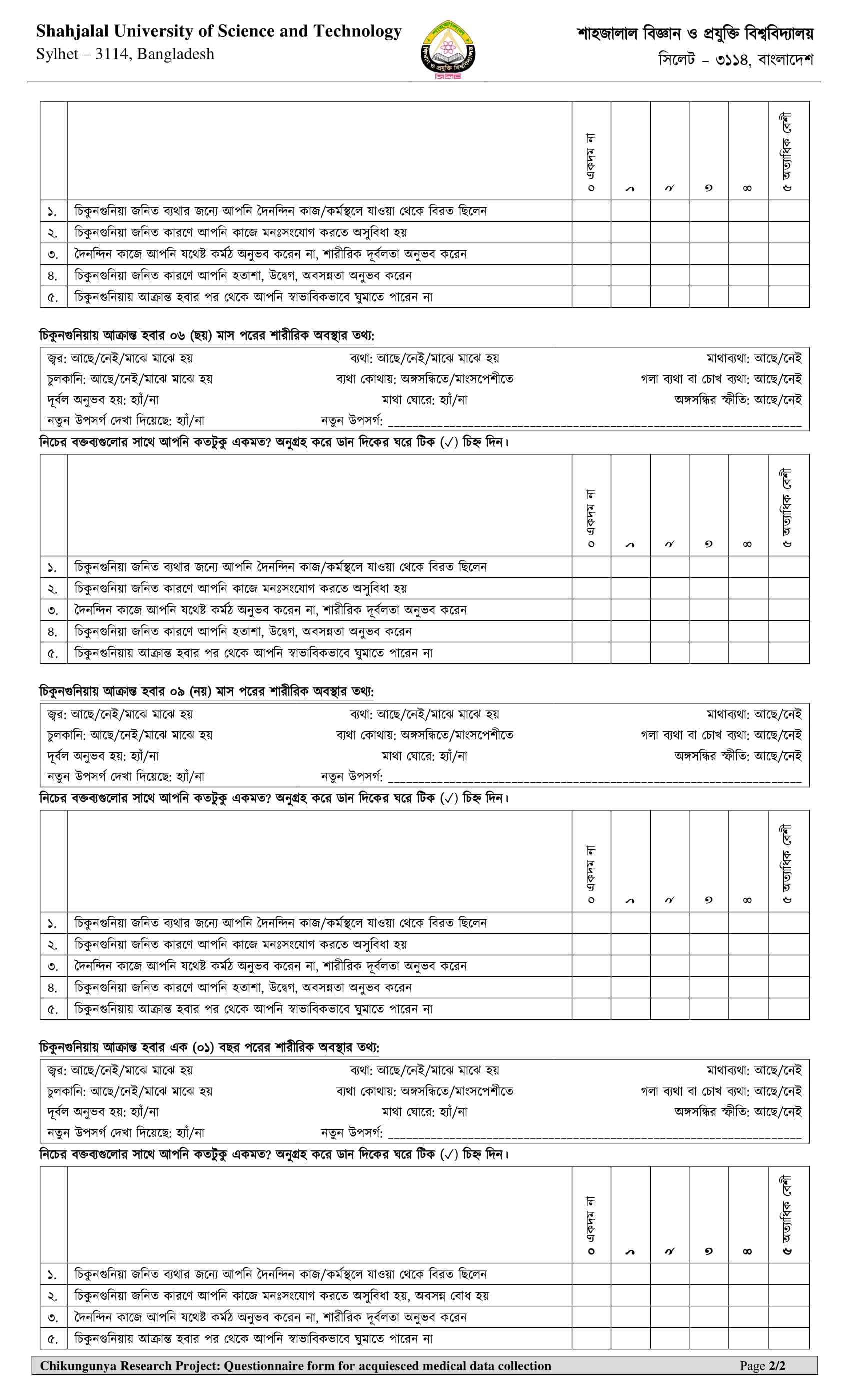

Supplement: S1 Fig — (DOCX) [file pntd.0007466.s002.docx]
