# Supplementary figures and images for: Chikungunya outbreak in Bangladesh (2017): Clinical and hematological findings
Source: PLoS Negl Trop Dis. 2020 Feb 24;14(2):e0007466. doi: 10.1371/journal.pntd.0007466 (PMC7058364; doi:10.1371/journal.pntd.0007466)

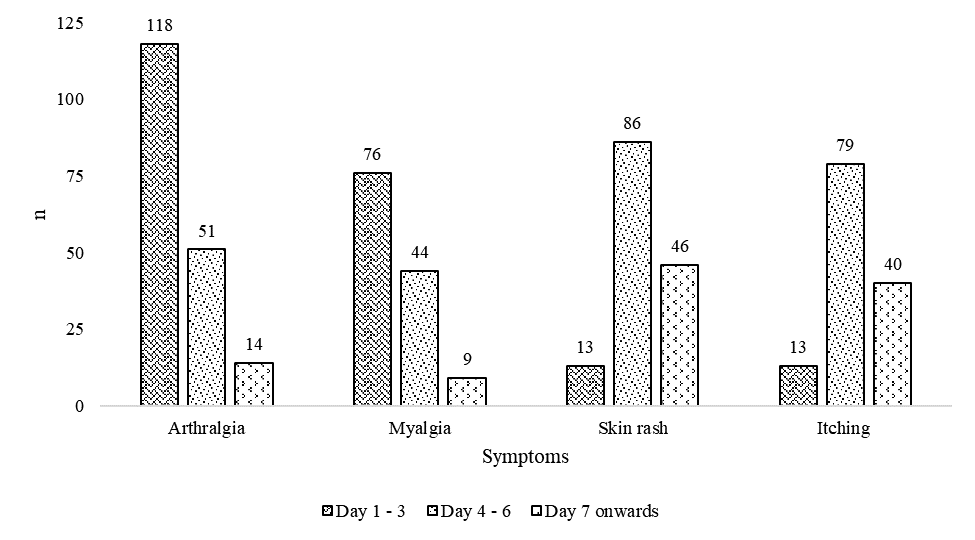


***

***

***

***

***S2 Fig. Onset of major symptoms during the acute phase of CHIKV infection.***

Supplement: S2 Fig — (DOCX) [file pntd.0007466.s003.docx]
