# Supplementary material for: Chikungunya outbreak in Bangladesh (2017): Clinical and hematological findings
Source: PLoS Negl Trop Dis. 2020 Feb 24;14(2):e0007466. doi: 10.1371/journal.pntd.0007466 (PMC7058364; doi:10.1371/journal.pntd.0007466)
Supplement: S3 Fig — (DOCX) [file pntd.0007466.s004.docx]

***S3 Fig. The intensity of arthralgia based on NRS****. The arthralgic intensity was categorized as mild (NR 1 – 4), moderate (NR 5 – 7) and severe (8 – 10). None of the patients reported mild arthralgia during the acute phase of the infection, while 85.5% of patients complained of severe pain.*
