# Supplementary material for: Chikungunya outbreak in Bangladesh (2017): Clinical and hematological findings
Source: PLoS Negl Trop Dis. 2020 Feb 24;14(2):e0007466. doi: 10.1371/journal.pntd.0007466 (PMC7058364; doi:10.1371/journal.pntd.0007466)
Supplement: S1 Table — (DOCX) [file pntd.0007466.s005.docx]

*S1 Table. Correlation between age and hematological data*

|  | | **Age** | **HB** | **ESR** | **RBC** | **WBC** | **PLAT** | **NTP** | **LPC** | **MC** | **EP** | **BP** | **HTC** | **MCV** | **MCH** | **MCHC** | **RDW** | **MPV** |
| --- | --- | --- | --- | --- | --- | --- | --- | --- | --- | --- | --- | --- | --- | --- | --- | --- | --- | --- |
| **Age** | Correlation (R) | 1 | -.070 | .137 | -.193^**^ | -.057 | -.096 | .173^*^ | -.157^*^ | -.004 | -.120 | .007 | -.147^*^ | .106 | .054 | -.121 | -.020 | -.132 |
|  | Sig. (2-tailed) |  | .342 | .062 | .009 | .439 | .192 | .018 | .032 | .953 | .103 | .921 | .047 | .151 | .467 | .102 | .863 | .267 |
| **HB** | Correlation (R) | -.070 | 1 | -.455^**^ | .584^**^ | -.104 | -.080 | .058 | -.084 | -.066 | .158^*^ | .165^*^ | .868^**^ | .464^**^ | .492^**^ | .396^**^ | -.555^**^ | .096 |
|  | Sig. (2-tailed) | .342 |  | .000 | .000 | .158 | .281 | .430 | .257 | .373 | .032 | .025 | .000 | .000 | .000 | .000 | .000 | .419 |
| **ESR** | Correlation (R) | .137 | -.455^**^ | 1 | -.374^**^ | .329^**^ | .190^**^ | .135 | -.113 | -.154^*^ | -.015 | -.072 | -.493^**^ | -.209^**^ | -.156^*^ | -.151^*^ | -.060 | -.041 |
|  | Sig. (2-tailed) | .062 | .000 |  | .000 | .000 | .009 | .066 | .126 | .036 | .837 | .330 | .000 | .004 | .034 | .041 | .613 | .731 |
| **RBC** | Correlation (R) | -.193^**^ | .584^**^ | -.374^**^ | 1 | -.053 | .034 | -.046 | .000 | .078 | .125 | .102 | .589^**^ | -.226^**^ | -.232^**^ | -.013 | .167 | -.066 |
|  | Sig. (2-tailed) | .009 | .000 | .000 |  | .474 | .649 | .533 | 1.000 | .288 | .090 | .166 | .000 | .002 | .002 | .863 | .154 | .581 |
| **WBC** | Correlation (R) | -.057 | -.104 | .329^**^ | -.053 | 1 | .578^**^ | .226^**^ | -.176^*^ | -.134 | -.156^*^ | -.036 | -.087 | -.091 | -.083 | -.006 | .113 | -.088 |
|  | Sig. (2-tailed) | .439 | .158 | .000 | .474 |  | .000 | .002 | .017 | .068 | .034 | .630 | .238 | .218 | .262 | .933 | .338 | .457 |
| **PLT** | Correlation (R) | -.096 | -.080 | .190^**^ | .034 | .578^**^ | 1 | .086 | -.040 | -.082 | -.120 | -.099 | -.088 | -.102 | -.101 | -.038 | .051 | -.202 |
|  | Sig. (2-tailed) | .192 | .281 | .009 | .649 | .000 |  | .245 | .589 | .267 | .105 | .180 | .237 | .167 | .175 | .612 | .669 | .087 |
| **NTP** | Correlation (R) | .173^*^ | .058 | .135 | -.046 | .226^**^ | .086 | 1 | -.954^**^ | -.104 | -.372^**^ | .049 | -.034 | .157^*^ | .120 | .183^*^ | -.140 | .128 |
|  | Sig. (2-tailed) | .018 | .430 | .066 | .533 | .002 | .245 |  | .000 | .159 | .000 | .509 | .647 | .033 | .106 | .013 | .235 | .280 |
| **LPC** | Correlation (R) | -.157^*^ | -.084 | -.113 | .000 | -.176^*^ | -.040 | -.954^**^ | 1 | -.081 | .158^*^ | -.082 | .008 | -.134 | -.082 | -.116 | .144 | -.140 |
|  | Sig. (2-tailed) | .032 | .257 | .126 | 1.000 | .017 | .589 | .000 |  | .274 | .031 | .267 | .913 | .070 | .267 | .117 | .222 | .238 |
| **MC** | Correlation (R) | -.004 | -.066 | -.154^*^ | .078 | -.134 | -.082 | -.104 | -.081 | 1 | -.068 | -.025 | .007 | -.150^*^ | -.135 | -.204^**^ | .018 | -.026 |
|  | Sig. (2-tailed) | .953 | .373 | .036 | .288 | .068 | .267 | .159 | .274 |  | .359 | .736 | .924 | .043 | .068 | .005 | .881 | .825 |
| **EP** | Correlation (R) | -.120 | .158^*^ | -.015 | .125 | -.156^*^ | -.120 | -.372^**^ | .158^*^ | -.068 | 1 | .132 | .105 | -.008 | -.061 | -.144 | -.003 | .076 |
|  | Sig. (2-tailed) | .103 | .032 | .837 | .090 | .034 | .105 | .000 | .031 | .359 |  | .074 | .155 | .915 | .414 | .050 | .978 | .522 |
| **BP** | Correlation (R) | .007 | .165^*^ | -.072 | .102 | -.036 | -.099 | .049 | -.082 | -.025 | .132 | 1 | .174^*^ | .085 | .084 | .011 | -.089 | .040 |
|  | Sig. (2-tailed) | .921 | .025 | .330 | .166 | .630 | .180 | .509 | .267 | .736 | .074 |  | .019 | .255 | .257 | .883 | .454 | .742 |
| **HTC** | Correlation (R) | -.147^*^ | .868^**^ | -.493^**^ | .589^**^ | -.087 | -.088 | -.034 | .008 | .007 | .105 | .174^*^ | 1 | .418^**^ | .406^**^ | .186^*^ | -.355^**^ | .114 |
|  | Sig. (2-tailed) | .047 | .000 | .000 | .000 | .238 | .237 | .647 | .913 | .924 | .155 | .019 |  | .000 | .000 | .011 | .002 | .335 |
| **MCV** | Correlation (R) | .106 | .464^**^ | -.209^**^ | -.226^**^ | -.091 | -.102 | .157^*^ | -.134 | -.150^*^ | -.008 | .085 | .418^**^ | 1 | .878^**^ | .398^**^ | -.547^**^ | .174 |
|  | Sig. (2-tailed) | .151 | .000 | .004 | .002 | .218 | .167 | .033 | .070 | .043 | .915 | .255 | .000 |  | .000 | .000 | .000 | .142 |
| **MCH** | Correlation (R) | .054 | .492^**^ | -.156^*^ | -.232^**^ | -.083 | -.101 | .120 | -.082 | -.135 | -.061 | .084 | .406^**^ | .878^**^ | 1 | .470^**^ | -.668^**^ | .092 |
|  | Sig. (2-tailed) | .467 | .000 | .034 | .002 | .262 | .175 | .106 | .267 | .068 | .414 | .257 | .000 | .000 |  | .000 | .000 | .437 |
| **MCHC** | Correlation (R) | -.121 | .396^**^ | -.151^*^ | -.013 | -.006 | -.038 | .183^*^ | -.116 | -.204^**^ | -.144 | .011 | .186^*^ | .398^**^ | .470^**^ | 1 | -.737^**^ | -.088 |
|  | Sig. (2-tailed) | .102 | .000 | .041 | .863 | .933 | .612 | .013 | .117 | .005 | .050 | .883 | .011 | .000 | .000 |  | .000 | .458 |
| **RDW** | Correlation (R) | -.020 | -.555^**^ | -.060 | .167 | .113 | .051 | -.140 | .144 | .018 | -.003 | -.089 | -.355^**^ | -.547^**^ | -.668^**^ | -.737^**^ | 1 | -.059 |
|  | Sig. (2-tailed) | .863 | .000 | .613 | .154 | .338 | .669 | .235 | .222 | .881 | .978 | .454 | .002 | .000 | .000 | .000 |  | .619 |
| **MPV** | Correlation (R) | -.132 | .096 | -.041 | -.066 | -.088 | -.202 | .128 | -.140 | -.026 | .076 | .040 | .114 | .174 | .092 | -.088 | -.059 | 1 |
|  | Sig. (2-tailed) | .267 | .419 | .731 | .581 | .457 | .087 | .280 | .238 | .825 | .522 | .742 | .335 | .142 | .437 | .458 | .619 |  |
| *Mean platelet volume = MPV; Red Cell Distribution Width = RDW; mean corpuscular hemoglobin concentration = MCHC; mean corpuscular hemoglobin = MCH; mean corpuscular volume = MCV; Hematocrit = HTC; Basophil = BP; Eosinophil = EP; Monocyte = MC* | | | | | | | | | | | | | | | | | | |
| ** Correlation is significant at the 0.01 level (2-tailed). | | | | | | | | | | | | | | | | | | |
| * Correlation is significant at the 0.05 level (2-tailed). | | | | | | | | | | | | | | | | | | |
