# Supplementary material for: Chikungunya outbreak in Bangladesh (2017): Clinical and hematological findings
Source: PLoS Negl Trop Dis. 2020 Feb 24;14(2):e0007466. doi: 10.1371/journal.pntd.0007466 (PMC7058364; doi:10.1371/journal.pntd.0007466)
Supplement: S2 Table — (DOCX) [file pntd.0007466.s006.docx]

***S2 Table.*** *Age stratified hematological findings in CHIKV positive patients.*

| **Age group (yrs)** | **Median** | **Range** | **Within**  **reference value**  **n (%)** | **Beyond**  **reference value**  **n (%)** | ***p* value^*^** |
| --- | --- | --- | --- | --- | --- |
| ***Hemoglobin level in g/dL (Reference range for male: 12 – 17 g/dL, female: 11.5 – 15.5 g/dL,*** ***children: 11 – 16 g/dL)*** | | | | | |
| Children (1 - 15) | 11.55 | 7.82 - 15.2 | 12 (37.5) | 20 (62.5) | 0.0158 |
| Young (16 - 25) | 13 | 9.5 - 15.6 | 19 (79.17) | 5 (20.83) | 0.0015 |
| Mid Aged (26 - 40) | 12.4 | 8.9 - 15.6 | 37 (63.79) | 21 (36.21) | 0.0027 |
| Seniors (41 - 59) | 12.1 | 8.1 - 14.9 | 27 (44.26) | 34 (55.74) | 0.0120 |
| Old (60^+^) | 10.55 | 9 - 13.2 | 5 (41.67) | 7 (58.33) | < 0.01 |
| **Total** |  |  | 100 (53.48) | 87 (46.52) |  |
| ***ESR in mm in 1st hour (Reference range for male: 0 - 10 mm, female: 0 - 20 mm, children: 0 – 10 mm)*** | | | | | |
| Children (1 – 15) | M: 23, F: 25 | 4 - 164 | 8, (25) | 24 (75) | < 0.01 |
| Young (16 - 25) | M: 30, F: 23 | 3 - 60 | 12 (50) | 12 (50) | < 0.01 |
| Mid Aged (26 - 40) | M: 12, F: 24 | 3 - 111 | 20 (34.48) | 38 (65.52) | < 0.01 |
| Seniors (41 - 59) | M: 13.5, F: 28 | 5 - 103 | 13 (21.31) | 48 (78.69) | < 0.01 |
| Old (60^+^) | M: 16.5, F: 31 | 12 - 101 | 1 (8.33) | 11 (91.67) | - |
| **Total** |  |  | 54 (28.88) | 133 (71.12) |  |
| ***RBC count in M/µL (Reference range for adult: 4.2 – 6.2 M/µL, children 4.0 – 5.5 M/µL)*** | | | | | |
| Children (1 - 15) | 4.4 | 3.7 - 5.66 | 27 (84.38) | 5 (15.62) | < 0.01 |
| Young (16 - 25) | 4.8 | 3.9 - 5.65 | 21 (87.50) | 3 (12.50) | < 0.01 |
| Mid Aged (26 - 40) | 4.6 | 3.8 - 6.4 | 11 (18.97) | 47 (81.03) | < 0.01 |
| Seniors (41 - 59) | 4.4 | 3.2 - 6.22 | 17 (27.87) | 44 (72.13) | < 0.01 |
| Old (60^+^) | 4.2 | 3.4 - 4.38 | 8 (75) | 4 (25) | 0.2969 |
| **Total** |  |  | 84 (44.92) | 103 (55.08) |  |
| ***WBC count in K/µL (Reference range for adult: 4.8 – 10.8 K/µL, children 4.8 – 10 K/µL)*** | | | | | |
| Children (1 - 15) | 6.725 | 4 - 12.2 | 17 (53.12) | 15 (46.88) | 0.8957 |
| Young (16 - 25) | 6 | 4.4 - 10.5 | 18 (75) | 6 (25) | < 0.01 |
| Mid Aged (26 - 40) | 6 | 3.7 - 12.5 | 39 (67.24) | 19 (32.75) | 0.9290 |
| Seniors (41 - 59) | 5.9 | 2 - 12.6 | 40 (65.57) | 21 (34.43) | 0.5306 |
| Old (60^+^) | 8 | 3 - 10.6 | 8 (75) | 4 (25) | < 0.01 |
| **Total** |  |  | 122 (65.24) | 65 (34.76) |  |
| ***Neutrophil part in % (Reference range: 40 – 70 %)*** | | | | | |
| Children (1 - 15) | 57.5 | 37 - 72 | 29 (90.63) | 3 (9.37 | 0.6752 |
| Young (16 - 25) | 61.5 | 32 - 80 | 14 (58.33) | 10 (41.67 | 0.2694 |
| Mid Aged (26 - 40) | 64 | 40 - 80 | 47 (81.03) | 11 (18.97 | 0.0053 |
| Seniors (41 - 59) | 62 | 52 - 75 | 58 (95.08) | 3 (4.92 | < 0.01 |
| Old (60^+^) | 66.5 | 46 - 75 | 10 (83.33) | 2 (16.67 | < 0.01 |
| **Total** |  |  | 158 (84.50) | 29 (15.50) |  |
| ***Lymphocyte part in % (Reference range: 20 – 45 %)*** | | | | | |
| Children (1 - 15) | 35 | 23 - 55 | 29 (90.63) | 3 (9.37) | < 0.01 |
| Young (16 - 25) | 27.5 | 15 - 52 | 18 (75) | 6 (25) | 0.9857 |
| Mid Aged (26 - 40) | 30 | 14 - 56 | 46 (79.31) | 12 (20.69) | 0.8713 |
| Seniors (41 - 59) | 33 | 17 - 42 | 60 (98.36) | 1 (1.64) | - |
| Old (60^+^) | 28 | 17 - 42 | 11 (91.67) | 1 (8.33) | - |
| **Total** |  |  | 164 (87.70) | 23 (12.29) |  |
| ***Platelets count in K/µL (Reference range for adult: 150 – 500 K/µL, children <10 yrs: 150 – 550 K/µL, children >10 yrs: 150 – 550 K/µL)*** | | | | | |
| Children (1 - 15) | 263.5 | 115 - 505 | 29 (90.63) | 3 (9.37 | 0.8831 |
| Young (16 - 25) | 226.5 | 135 - 515 | 21 (87.5) | 3 (12.5 | 0.8713 |
| Mid Aged (26 - 40) | 229 | 150 - 547 | 56 (96.55) | 2 (3.45 | < 0.01 |
| Seniors (41 - 59) | 255 | 85 - 465 | 59 (96.72) | 2 (3.28 | < 0.01 |
| Old (60^+^) | 242.5 | 168 - 372 | 12 (100) | 0 (0) | - |
| **Total** |  |  | 177 (94.65) | 10 (5.35) |  |

*M = Male, F = Female.     ^*^Calculated from z score*.
