# Supplementary material for: Chikungunya outbreak in Bangladesh (2017): Clinical and hematological findings
Source: PLoS Negl Trop Dis. 2020 Feb 24;14(2):e0007466. doi: 10.1371/journal.pntd.0007466 (PMC7058364; doi:10.1371/journal.pntd.0007466)
Supplement: S3 Table — A: LCA study. Demographic information of the patients as well as the presence of fever and joint pain at different time points. B: Outcomes of McNemar test. C: Number of patients visited a physician due to post-CHIKV complications (n = 48). (DOCX) [file pntd.0007466.s007.docx]

**S3 Table A: LCA study.** *Demographic information of the patients as well as the presence of fever and joint pain at different time points.*

| Sl. | Registration | Gender | Fever | Sites of arthralgia | | | | | | | | | | | |  |
| --- | --- | --- | --- | --- | --- | --- | --- | --- | --- | --- | --- | --- | --- | --- | --- | --- |
|  |  |  |  | Finger | Wrist | Back | Waist | Knee | Armpit | Rib Joints | Shoulder & Neck | Elbow | Thigh | Ankle | Feet |  |
| 1 | UT001  Age: 50 yrs  Occupation: Security guard  Education Level: Primary  Economic Status: Low income | M | + | + | + |  | + | + | + | + |  | + |  | + |  | Day 0 - 7 |
|  |  |  |  |  |  |  |  |  |  |  |  |  |  |  |  | M2 |
|  |  |  |  |  |  |  |  |  |  |  |  |  |  |  |  | M4 |
|  |  |  |  |  |  |  |  |  |  |  |  |  |  |  |  | M6 |
|  |  |  |  |  |  |  |  |  |  |  |  |  |  |  |  | M9 |
|  |  |  |  |  |  |  |  |  |  |  |  |  |  |  |  | M12 |
| 2 | UT002  Age: 46 yrs  Occupation: Housewife  Education Level: Primary  Economic Status: Middle class | F | + | + | + | + | + | + |  | + | + | + | + | + |  | Day 0 - 7 |
|  |  |  |  |  |  |  |  |  |  |  |  |  |  |  |  | M2 |
|  |  |  |  |  |  |  |  |  |  |  |  |  |  |  |  | M4 |
|  |  |  |  |  |  |  |  |  |  |  |  |  |  |  |  | M6 |
|  |  |  |  |  |  |  |  |  |  |  |  |  |  |  |  | M9 |
|  |  |  |  |  |  |  |  |  |  |  |  |  |  |  |  | M12 |
| 3 | UT003  Age: 20 yrs  Occupation: Student  Education Level: Bachelors (ongoing)  Economic Status: Middle income | F | + | + | + |  | + | + |  | + |  |  |  | + |  | Day 0 - 7 |
|  |  |  |  |  |  |  |  |  |  |  |  |  |  |  |  | M2 |
|  |  |  |  |  |  |  |  |  |  |  |  |  |  |  |  | M4 |
|  |  |  |  |  |  |  |  |  |  |  |  |  |  |  |  | M6 |
|  |  |  |  |  |  |  |  |  |  |  |  |  |  |  |  | M9 |
|  |  |  |  |  |  |  |  |  |  |  |  |  |  |  |  | M12 |
| 4 | UT004  Age: 35 yrs  Occupation: Teacher  Education Level: Postgraduate  Economic Status: High income | F | + | + | + |  |  | + | + | + | + |  |  |  |  | Day 0 - 7 |
|  |  |  |  |  |  |  |  |  |  |  |  |  |  |  |  | M2 |
|  |  |  |  |  |  |  |  |  |  |  |  |  |  |  |  | M4 |
|  |  |  |  |  |  |  |  |  |  |  |  |  |  |  |  | M6 |
|  |  |  |  |  |  |  |  |  |  |  |  |  |  |  |  | M9 |
|  |  |  |  |  |  |  |  |  |  |  |  |  |  |  |  | M12 |

| 5 | MT001  Age: 48 yrs  Occupation: Housewife  Education Level: Secondary  Economic Status: Low income | F | + | + | + |  | + | + |  |  |  |  |  | + | + | Day 0 - 7 |
| --- | --- | --- | --- | --- | --- | --- | --- | --- | --- | --- | --- | --- | --- | --- | --- | --- |
|  |  |  |  | + | + |  |  | + |  |  |  |  |  | + | + | M2 |
|  |  |  |  |  |  |  |  |  |  |  |  |  |  |  |  | M4 |
|  |  |  |  |  |  |  |  |  |  |  |  |  |  |  |  | M6 |
|  |  |  |  |  |  |  |  |  |  |  |  |  |  |  |  | M9 |
|  |  |  |  |  |  |  |  |  |  |  |  |  |  |  |  | M12 |
| 6 | UT005  Age: 23 yrs  Occupation: Brick labour  Education Level: Primary  Economic Status: Low income | M | + | + | + | + | + | + | + | + | + | + | + | + | + | Day 0 - 7 |
|  |  |  |  |  |  |  |  |  |  |  |  |  |  |  |  | M2 |
|  |  |  |  |  |  |  |  |  |  |  |  |  |  |  |  | M4 |
|  |  |  |  |  |  |  |  |  |  |  |  |  |  |  |  | M6 |
|  |  |  |  |  |  |  |  |  |  |  |  |  |  |  |  | M9 |
|  |  |  |  |  |  |  |  |  |  |  |  |  |  |  |  | M12 |
| 7 | UT006  Age: 38 yrs  Occupation: Electrician  Education Level: Higher secondary  Economic Status: Low income | M | + | + | + |  | + |  |  | + |  |  |  |  |  | Day 0 - 7 |
|  |  |  | + | + | + |  | + |  |  |  |  |  |  |  |  | M2 |
|  |  |  |  | + | + |  |  |  |  |  |  |  |  |  |  | M4 |
|  |  |  |  |  |  |  |  |  |  |  |  |  |  |  |  | M6 |
|  |  |  |  | + | + |  |  |  |  |  |  |  |  | + |  | M9 |
|  |  |  |  | + | + |  |  |  |  |  |  |  |  |  |  | M12 |
| 8 | DH001  Age: 36 yrs  Occupation: Housewife  Education Level: Graduate  Economic Status: High income | F | + | + | + |  | + | + | + |  |  |  |  | + |  | Day 0 - 7 |
|  |  |  |  |  |  |  |  |  |  |  |  |  |  |  |  | M2 |
|  |  |  |  |  |  |  |  |  |  |  |  |  |  |  |  | M4 |
|  |  |  |  |  |  |  |  |  |  |  |  |  |  |  |  | M6 |
|  |  |  |  |  |  |  |  |  |  |  |  |  |  |  |  | M9 |
|  |  |  |  |  |  |  |  |  |  |  |  |  |  |  |  | M12 |
| 9 | UT007  Age: 26 yrs  Occupation: Student  Education Level: Postgraduate (ongoing)  Economic Status: Middle income | F | + | + | + |  | + | + |  | + |  |  |  | + |  | Day 0 - 7 |
|  |  |  | + | + | + |  |  | + |  |  |  |  |  | + |  | M2 |
|  |  |  |  |  |  |  |  |  |  |  |  |  |  |  |  | M4 |
|  |  |  |  |  |  |  |  |  |  |  |  |  |  |  |  | M6 |
|  |  |  |  |  |  |  |  |  |  |  |  |  |  |  |  | M9 |
|  |  |  |  |  |  |  |  |  |  |  |  |  |  |  |  | M12 |
| 10 | DK002  Age: 34 yrs  Occupation: Housewife  Education Level: Higher secondary  Economic Status: High income | F | + | + | + | + | + | + | + |  |  |  |  | + | + | Day 0 - 7 |
|  |  |  |  |  |  |  |  |  |  |  |  |  |  |  |  | M2 |
|  |  |  |  |  |  |  |  |  |  |  |  |  |  |  |  | M4 |
|  |  |  |  |  |  |  |  |  |  |  |  |  |  |  |  | M6 |
|  |  |  |  |  |  |  |  |  |  |  |  |  |  |  |  | M9 |
|  |  |  |  |  |  |  |  |  |  |  |  |  |  |  |  | M12 |
| 11 | UT008  Age: 47 yrs  Occupation: Service holder  Education Level: Bachelors  Economic Status: High income | M | + | + | + |  |  | + |  | + |  |  |  | + | + | Day 0 - 7 |
|  |  |  |  |  |  |  |  |  |  |  |  |  |  |  |  | M2 |
|  |  |  |  |  |  |  |  |  |  |  |  |  |  |  |  | M4 |
|  |  |  |  |  |  |  |  |  |  |  |  |  |  |  |  | M6 |
|  |  |  |  |  |  |  |  |  |  |  |  |  |  |  |  | M9 |
|  |  |  |  |  |  |  |  |  |  |  |  |  |  |  |  | M12 |
| 12 | MY001  Age: 34 yrs  Occupation: Housewife  Education Level: Bachelors  Economic Status: High income | F | + | + | + |  | + | + |  |  |  |  |  | + | + | Day 0 - 7 |
|  |  |  | + | + |  |  |  | + |  |  |  |  |  | + | + | M2 |
|  |  |  |  | + |  |  |  |  |  |  |  |  |  | + |  | M4 |
|  |  |  |  |  |  |  |  |  |  |  |  |  |  |  |  | M6 |
|  |  |  |  |  |  |  |  |  |  |  |  |  |  |  |  | M9 |
|  |  |  |  |  |  |  |  |  |  |  |  |  |  |  |  | M12 |
| 13 | MT002  Age: 47 yrs  Occupation: Housewife  Education Level: Bachelors  Economic Status: High income | F | + | + | + |  |  | + |  |  |  |  |  | + |  | Day 0 - 7 |
|  |  |  |  |  |  |  |  |  |  |  |  |  |  |  |  | M2 |
|  |  |  |  |  |  |  |  |  |  |  |  |  |  |  |  | M4 |
|  |  |  |  |  |  |  |  |  |  |  |  |  |  |  |  | M6 |
|  |  |  |  |  |  |  |  |  |  |  |  |  |  |  |  | M9 |
|  |  |  |  |  |  |  |  |  |  |  |  |  |  |  |  | M12 |
| 14 | DK003  Age: 34 yrs  Occupation: Service holder  Education Level: Post graduation  Economic Status:High income | M | + | + | + |  |  | + |  |  |  |  | + | + | + | Day 0 - 7 |
|  |  |  |  |  |  |  |  |  |  |  |  |  |  |  |  | M2 |
|  |  |  |  |  |  |  |  |  |  |  |  |  |  |  |  | M4 |
|  |  |  |  |  |  |  |  |  |  |  |  |  |  |  |  | M6 |
|  |  |  |  |  |  |  |  |  |  |  |  |  |  |  |  | M9 |
|  |  |  |  |  |  |  |  |  |  |  |  |  |  |  |  | M12 |
| 15 | UT009  Age: 46 yrs  Occupation: Business  Education Level: Bachelors  Economic Status: Middle income | M | + | + | + | + | + |  |  | + | + |  |  | + | + | Day 0 - 7 |
|  |  |  |  |  |  |  |  |  |  |  |  |  |  |  |  | M2 |
|  |  |  |  |  |  |  |  |  |  |  |  |  |  |  |  | M4 |
|  |  |  |  |  |  |  |  |  |  |  |  |  |  |  |  | M6 |
|  |  |  |  |  |  |  |  |  |  |  |  |  |  |  |  | M9 |
|  |  |  |  |  |  |  |  |  |  |  |  |  |  |  |  | M12 |
| 16 | UT010  Age: 27 yrs  Occupation: Service holder  Education Level: Post graduation  Economic Status: Middle income | F | + | + | + |  | + | + |  | + |  |  | + | + | + | Day 0 - 7 |
|  |  |  | + |  |  |  |  |  |  |  |  |  |  |  |  | M2 |
|  |  |  |  |  |  |  |  |  |  |  |  |  |  |  |  | M4 |
|  |  |  |  |  |  |  |  |  |  |  |  |  |  |  |  | M6 |
|  |  |  |  |  |  |  |  |  |  |  |  |  |  |  |  | M9 |
|  |  |  |  |  |  |  |  |  |  |  |  |  |  |  |  | M12 |
| 17 | DK004  Age: 46 yrs  Occupation: Secondary  Education Level: Business  Economic Status: Middle income | F | + | + | + |  | + |  |  |  | + |  | + | + | + | Day 0 - 7 |
|  |  |  |  |  |  |  |  |  |  |  |  |  |  |  |  | M2 |
|  |  |  |  |  |  |  |  |  |  |  |  |  |  |  |  | M4 |
|  |  |  |  |  |  |  |  |  |  |  |  |  |  |  |  | M6 |
|  |  |  |  |  |  |  |  |  |  |  |  |  |  |  |  | M9 |
|  |  |  |  |  |  |  |  |  |  |  |  |  |  |  |  | M12 |
| 18 | MY002  Age: 28 yrs  Occupation: Graphic designer  Education Level: Post graduation  Economic Status: Middle income | M | + | + | + |  | + | + |  |  | + |  | + | + | + | Day 0 - 7 |
|  |  |  |  |  |  |  |  |  |  |  |  |  |  |  |  | M2 |
|  |  |  |  |  |  |  |  |  |  |  |  |  |  |  |  | M4 |
|  |  |  |  |  |  |  |  |  |  |  |  |  |  |  |  | M6 |
|  |  |  |  |  |  |  |  |  |  |  |  |  |  |  |  | M9 |
|  |  |  |  |  |  |  |  |  |  |  |  |  |  |  |  | M12 |
| 19 | MT003  Age: 33 yrs  Occupation: Housewife  Education Level: Graduation  Economic Status: Middle income | F | + | + |  |  | + | + |  |  |  |  |  | + |  | Day 0 - 7 |
|  |  |  |  |  |  |  |  |  |  |  |  |  |  |  |  | M2 |
|  |  |  |  |  |  |  |  |  |  |  |  |  |  |  |  | M4 |
|  |  |  |  |  |  |  |  |  |  |  |  |  |  |  |  | M6 |
|  |  |  |  |  |  |  |  |  |  |  |  |  |  |  |  | M9 |
|  |  |  |  |  |  |  |  |  |  |  |  |  |  |  |  | M12 |
| 20 | DK005  Age: 52 yrs  Occupation: Service holder  Education Level: Bachelors  Economic Status: High income | M | + | + | + | + | + | + |  |  |  |  | + | + | + | Day 0 - 7 |
|  |  |  |  |  |  |  |  |  |  |  |  |  |  |  |  | M2 |
|  |  |  |  |  |  |  |  |  |  |  |  |  |  |  |  | M4 |
|  |  |  |  |  |  |  |  |  |  |  |  |  |  |  |  | M6 |
|  |  |  |  |  |  |  |  |  |  |  |  |  |  |  |  | M9 |
|  |  |  |  |  |  |  |  |  |  |  |  |  |  |  |  | M12 |
| 21 | MY003  Age: 32 yrs  Occupation: Service holder  Education Level: Post graduation  Economic Status: High income | F | + | + |  |  |  | + |  |  |  |  |  | + | + | Day 0 - 7 |
|  |  |  |  |  |  |  |  |  |  |  |  |  |  |  |  | M2 |
|  |  |  |  |  |  |  |  |  |  |  |  |  |  |  |  | M4 |
|  |  |  |  |  |  |  |  |  |  |  |  |  |  |  |  | M6 |
|  |  |  |  |  |  |  |  |  |  |  |  |  |  |  |  | M9 |
|  |  |  |  |  |  |  |  |  |  |  |  |  |  |  |  | M12 |
| 22 | DK006  Age: 40 yrs  Occupation: Teacher  Education Level: Post graduation  Economic Status: High income | M | + | + | + |  |  | + |  |  | + |  |  | + | + | Day 0 - 7 |
|  |  |  |  | + | + |  |  |  |  |  | + |  |  | + | + | M2 |
|  |  |  |  | + | + |  |  |  |  |  |  |  |  |  |  | M4 |
|  |  |  |  | + | + |  |  | + |  |  |  |  |  | + | + | M6 |
|  |  |  |  | + | + |  |  |  |  |  |  |  |  | + |  | M9 |
|  |  |  |  | + | + |  |  |  |  |  |  |  |  |  |  | M12 |
| 23 | DK007  Age: 3+ yrs  Occupation: Driver  Education Level: Primary  Economic Status: Middle income | M | + | + | + |  | + | + |  |  |  |  |  | + | + | Day 0 - 7 |
|  |  |  | + | + |  |  |  | + |  |  |  |  |  | + | + | M2 |
|  |  |  |  |  |  |  |  |  |  |  |  |  |  |  |  | M4 |
|  |  |  |  |  |  |  |  |  |  |  |  |  |  |  |  | M6 |
|  |  |  |  |  |  |  |  |  |  |  |  |  |  |  |  | M9 |
|  |  |  |  |  |  |  |  |  |  |  |  |  |  |  |  | M12 |
| 24 | DK008  Age: 23 yrs  Occupation: Student  Education Level: Bachelors (ongoing)  Economic Status: Middle income | M | + | + | + | + |  | + |  |  |  |  |  | + | + | Day 0 - 7 |
|  |  |  |  | + |  |  |  | + |  |  |  |  |  | + | + | M2 |
|  |  |  |  |  |  |  |  |  |  |  |  |  |  |  |  | M4 |
|  |  |  |  |  |  |  |  |  |  |  |  |  |  |  |  | M6 |
|  |  |  |  |  |  |  |  |  |  |  |  |  |  |  |  | M9 |
|  |  |  |  |  |  |  |  |  |  |  |  |  |  |  |  | M12 |
| 25 | MY004  Age: 22 yrs  Occupation: Student  Education Level: Bachelors (ongoing)  Economic Status: High income | F | + | + | + |  |  | + |  |  | + |  |  | + | + | Day 0 - 7 |
|  |  |  |  |  |  |  |  |  |  |  |  |  |  |  |  | M2 |
|  |  |  |  |  |  |  |  |  |  |  |  |  |  |  |  | M4 |
|  |  |  |  |  |  |  |  |  |  |  |  |  |  |  |  | M6 |
|  |  |  |  |  |  |  |  |  |  |  |  |  |  |  |  | M9 |
|  |  |  |  |  |  |  |  |  |  |  |  |  |  |  |  | M12 |
| 26 | DK009  Age: 22 yrs  Occupation: Security superintendent  Education Level: Secondary  Economic Status: Middle income | M | + | + | + |  | + | + |  |  | + |  |  | + | + | Day 0 - 7 |
|  |  |  |  | + | + |  |  | + |  |  |  |  |  | + | + | M2 |
|  |  |  |  | + | + |  |  |  |  |  |  |  |  | + | + | M4 |
|  |  |  |  | + |  |  |  |  |  |  |  |  |  | + | + | M6 |
|  |  |  |  | + |  |  |  |  |  |  |  |  |  |  |  | M9 |
|  |  |  |  | + | + |  |  |  |  |  |  |  |  |  |  | M12 |
| 27 | MT004  Age: +8 yrs  Occupation: Student  Education Level: Higher secondary (ongoing)  Economic Status: Middle income | M | + | + | + |  | + | + |  |  | + |  |  |  |  | Day 0 - 7 |
|  |  |  |  |  |  |  |  |  |  |  |  |  |  |  |  | M2 |
|  |  |  |  |  |  |  |  |  |  |  |  |  |  |  |  | M4 |
|  |  |  |  |  |  |  |  |  |  |  |  |  |  |  |  | M6 |
|  |  |  |  |  |  |  |  |  |  |  |  |  |  |  |  | M9 |
|  |  |  |  |  |  |  |  |  |  |  |  |  |  |  |  | M12 |
| 28 | UT011  Age: 42 yrs  Occupation: Housewife  Education Level: Bachelors  Economic Status: High income | F | + | + | + | + | + | + |  | + | + | + |  | + | + | Day 0 - 7 |
|  |  |  | + | + | + |  | + | + |  |  |  |  |  | + | + | M2 |
|  |  |  | + | + | + |  |  | + |  |  |  |  |  | + | + | M4 |
|  |  |  |  | + |  |  |  |  |  |  |  |  |  |  |  | M6 |
|  |  |  |  | + |  |  |  |  |  |  |  |  |  |  |  | M9 |
|  |  |  |  | + | + |  |  |  |  |  |  |  |  |  |  | M12 |
| 29 | MY005  Age: 35 yrs  Occupation: Media personnel  Education Level: Post graduation  Economic Status: High income | M | + | + | + |  | + |  |  |  | + |  |  | + | + | Day 0 - 7 |
|  |  |  |  |  |  |  |  |  |  |  |  |  |  |  |  | M2 |
|  |  |  |  |  |  |  |  |  |  |  |  |  |  |  |  | M4 |
|  |  |  |  |  |  |  |  |  |  |  |  |  |  |  |  | M6 |
|  |  |  |  |  |  |  |  |  |  |  |  |  |  |  |  | M9 |
|  |  |  |  |  |  |  |  |  |  |  |  |  |  |  |  | M12 |
| 30 | MY006  Age: 2+ yrs  Occupation: Student  Education Level: Higher secondary  Economic Status: Low income | M | + | + | + |  | + |  |  |  |  |  |  | + | + | Day 0 - 7 |
|  |  |  |  |  |  |  |  |  |  |  |  |  |  |  |  | M2 |
|  |  |  |  |  |  |  |  |  |  |  |  |  |  |  |  | M4 |
|  |  |  |  |  |  |  |  |  |  |  |  |  |  |  |  | M6 |
|  |  |  |  |  |  |  |  |  |  |  |  |  |  |  |  | M9 |
|  |  |  |  |  |  |  |  |  |  |  |  |  |  |  |  | M12 |
| 31 | DK010  Age: 24 yrs  Occupation: Student  Education Level: Bachelors (Ongoing)  Economic Status: High income | F | + | + | + |  |  | + |  |  |  |  |  | + | + | Day 0 - 7 |
|  |  |  | + |  |  |  |  |  |  |  |  |  |  |  |  | M2 |
|  |  |  |  |  |  |  |  |  |  |  |  |  |  |  |  | M4 |
|  |  |  |  |  |  |  |  |  |  |  |  |  |  |  |  | M6 |
|  |  |  |  |  |  |  |  |  |  |  |  |  |  |  |  | M9 |
|  |  |  |  |  |  |  |  |  |  |  |  |  |  |  |  | M12 |
| 32 | DK011  Age: 50 yrs  Occupation: Business  Education Level: Secondary  Economic Status: Middle income | M | + | + | + |  |  | + | + |  |  |  |  | + | + | Day 0 - 7 |
|  |  |  | + | + | + |  |  | + |  |  |  |  |  | + | + | M2 |
|  |  |  | + | + |  |  |  |  |  |  |  |  |  | + | + | M4 |
|  |  |  |  |  |  |  |  |  |  |  |  |  |  |  |  | M6 |
|  |  |  |  | + |  |  |  |  |  |  |  |  |  | + |  | M9 |
|  |  |  |  | + | + |  |  |  |  |  |  |  |  | + |  | M12 |
| 33 | MY007  Age: 24 yrs  Occupation: Student  Education Level: Bachelors (Ongoing)  Economic Status: Middle income | M | + | + | + |  | + | + |  |  |  |  |  | + | + | Day 0 - 7 |
|  |  |  |  | + |  |  |  |  |  |  |  |  |  | + | + | M2 |
|  |  |  |  |  |  |  |  |  |  |  |  |  |  |  |  | M4 |
|  |  |  |  |  |  |  |  |  |  |  |  |  |  |  |  | M6 |
|  |  |  |  |  |  |  |  |  |  |  |  |  |  |  |  | M9 |
|  |  |  |  |  |  |  |  |  |  |  |  |  |  |  |  | M12 |
| 34 | DK012  Age: 27 yrs  Occupation: Engineer  Education Level: Post graduation  Economic Status: High income | F | + | + | + |  | + | + |  |  | + |  |  | + | + | Day 0 - 7 |
|  |  |  |  | + |  |  |  |  |  |  |  |  |  |  |  | M2 |
|  |  |  |  |  |  |  |  |  |  |  |  |  |  |  |  | M4 |
|  |  |  |  |  |  |  |  |  |  |  |  |  |  |  |  | M6 |
|  |  |  |  |  |  |  |  |  |  |  |  |  |  |  |  | M9 |
|  |  |  |  |  |  |  |  |  |  |  |  |  |  |  |  | M12 |
| 35 | DK013  Age: 24 yrs  Occupation: Student  Education Level: Post graduation (Ongoing)  Economic Status: High income | F | + | + | + |  | + | + |  |  |  |  | + | + | + | Day 0 - 7 |
|  |  |  |  |  |  |  |  |  |  |  |  |  |  |  |  | M2 |
|  |  |  |  | + | + |  |  |  |  |  |  |  |  |  |  | M4 |
|  |  |  |  |  |  |  |  |  |  |  |  |  |  |  |  | M6 |
|  |  |  |  |  |  |  |  |  |  |  |  |  |  |  |  | M9 |
|  |  |  |  |  |  |  |  |  |  |  |  |  |  |  |  | M12 |
| 36 | MY008  Age: 24 yrs  Occupation: Student  Education Level: Bachelors (ongoing)  Economic Status: High income | M | + | + |  |  |  |  |  |  |  |  |  |  | + | Day 0 - 7 |
|  |  |  | + |  |  |  |  |  |  |  |  |  |  |  |  | M2 |
|  |  |  |  |  |  |  |  |  |  |  |  |  |  |  |  | M4 |
|  |  |  |  |  |  |  |  |  |  |  |  |  |  |  |  | M6 |
|  |  |  |  |  |  |  |  |  |  |  |  |  |  |  |  | M9 |
|  |  |  |  |  |  |  |  |  |  |  |  |  |  |  |  | M12 |
| 37 | MT005  Age: 25 yrs  Occupation: Business (grocery shop)  Education Level: Primary  Economic Status: Low income | M | + | + | + |  | + | + |  |  |  |  |  | + |  | Day 0 - 7 |
|  |  |  |  |  |  |  |  |  |  |  |  |  |  |  |  | M2 |
|  |  |  |  |  |  |  |  |  |  |  |  |  |  |  |  | M4 |
|  |  |  |  |  |  |  |  |  |  |  |  |  |  |  |  | M6 |
|  |  |  |  |  |  |  |  |  |  |  |  |  |  |  |  | M9 |
|  |  |  |  |  |  |  |  |  |  |  |  |  |  |  |  | M12 |
| 38 | MY009  Age: 45 yrs  Occupation: Business  Education Level: Bachelors  Economic Status: High income | M | + | + | + |  |  | + |  |  |  |  |  | + | + | Day 0 - 7 |
|  |  |  |  |  |  |  |  |  |  |  |  |  |  |  |  | M2 |
|  |  |  |  |  |  |  |  |  |  |  |  |  |  |  |  | M4 |
|  |  |  |  |  |  |  |  |  |  |  |  |  |  |  |  | M6 |
|  |  |  |  |  |  |  |  |  |  |  |  |  |  |  |  | M9 |
|  |  |  |  |  |  |  |  |  |  |  |  |  |  |  |  | M12 |
| 39 | DK014  Age: 36 yrs  Occupation: Housewife  Education Level: Secondary  Economic Status: Low income | F | + | + | + |  | + | + |  |  |  |  |  | + | + | Day 0 - 7 |
|  |  |  |  | + | + |  |  |  |  |  |  |  |  | + | + | M2 |
|  |  |  |  | + | + |  |  |  |  |  |  |  |  | + | + | M4 |
|  |  |  |  | + |  |  | + |  |  |  |  |  |  |  |  | M6 |
|  |  |  |  | + |  |  | + |  |  |  |  |  |  |  |  | M9 |
|  |  |  |  | + |  |  |  |  |  |  |  |  |  |  |  | M12 |
| 40 | DK015  Age: 34 yrs  Occupation: Engineer  Education Level: Bachelors  Economic Status: Middle income | M | + | + | + |  | + | + |  |  |  |  | + | + | + | Day 0 - 7 |
|  |  |  |  | + | + |  |  |  |  |  |  |  |  | + | + | M2 |
|  |  |  |  | + | + |  |  |  |  |  |  |  |  | + | + | M4 |
|  |  |  |  | + |  |  |  |  |  |  |  |  |  |  |  | M6 |
|  |  |  |  | + |  |  |  |  |  |  |  |  |  |  |  | M9 |
|  |  |  |  | + |  |  |  |  |  |  |  |  |  |  |  | M12 |
| 41 | MY010  Age: 27 yrs  Occupation: Business  Education Level: Higher secondary  Economic Status: Middle income | M | + | + | + |  | + |  |  |  | + |  | + | + | + | Day 0 - 7 |
|  |  |  |  |  |  |  |  |  |  |  |  |  |  |  |  | M2 |
|  |  |  |  |  |  |  |  |  |  |  |  |  |  |  |  | M4 |
|  |  |  |  |  |  |  |  |  |  |  |  |  |  |  |  | M6 |
|  |  |  |  |  |  |  |  |  |  |  |  |  |  |  |  | M9 |
|  |  |  |  |  |  |  |  |  |  |  |  |  |  |  |  | M12 |
| 42 | MY011  Age: 29 yrs  Occupation: Service holder  Education Level: Post graduation  Economic Status: High income | F | + | + | + |  | + |  |  |  |  |  |  | + | + | Day 0 - 7 |
|  |  |  | + |  |  |  |  |  |  |  |  |  |  |  |  | M2 |
|  |  |  |  |  |  |  |  |  |  |  |  |  |  |  |  | M4 |
|  |  |  |  |  |  |  |  |  |  |  |  |  |  |  |  | M6 |
|  |  |  |  |  |  |  |  |  |  |  |  |  |  |  |  | M9 |
|  |  |  |  |  |  |  |  |  |  |  |  |  |  |  |  | M12 |
| 43 | DK016  Age: 5+ yrs  Occupation: Rickshaw/van puller  Education Level:  Economic Status: Low income | M | + | + | + |  | + | + |  |  | + |  |  | + | + | Day 0 - 7 |
|  |  |  |  |  |  |  |  |  |  |  |  |  |  |  |  | M2 |
|  |  |  |  |  |  |  |  |  |  |  |  |  |  |  |  | M4 |
|  |  |  |  |  |  |  |  |  |  |  |  |  |  |  |  | M6 |
|  |  |  |  |  |  |  |  |  |  |  |  |  |  |  |  | M9 |
|  |  |  |  |  |  |  |  |  |  |  |  |  |  |  |  | M12 |
| 44 | MY012  Age: 25 yrs  Occupation: Plumber  Education Level: Secondary  Economic Status: Middle income | M | + | + | + |  |  |  |  |  |  |  | + | + | + | Day 0 - 7 |
|  |  |  |  | + | + |  |  |  |  |  |  |  |  | + | + | M2 |
|  |  |  |  | + | + |  |  |  |  |  |  |  |  | + |  | M4 |
|  |  |  |  | + |  |  |  |  |  |  |  |  |  |  |  | M6 |
|  |  |  |  | + |  |  |  |  |  |  |  |  |  |  |  | M9 |
|  |  |  |  | + |  |  |  |  |  |  |  |  |  | + |  | M12 |
| 45 | DK017  Age: 4+ yrs  Occupation: Bus driver  Education Level: Secondary  Economic Status: Middle income | M | + | + | + |  | + | + |  |  | + |  |  | + | + | Day 0 - 7 |
|  |  |  |  | + |  |  |  |  |  |  |  |  |  | + | + | M2 |
|  |  |  |  |  |  |  |  |  |  |  |  |  |  |  |  | M4 |
|  |  |  |  |  |  |  |  |  |  |  |  |  |  |  |  | M6 |
|  |  |  |  |  |  |  |  |  |  |  |  |  |  |  |  | M9 |
|  |  |  |  |  |  |  |  |  |  |  |  |  |  |  |  | M12 |
| 46 | DK018  Age: 34 yrs  Occupation: Housewife  Education Level: Secondary  Economic Status: Low income | F | + | + |  | + | + |  |  |  | + |  | + | + | + | Day 0 - 7 |
|  |  |  |  | + |  |  |  |  |  |  |  |  | + | + | + | M2 |
|  |  |  |  |  |  |  |  |  |  |  |  |  |  |  |  | M4 |
|  |  |  |  |  |  |  |  |  |  |  |  |  |  |  |  | M6 |
|  |  |  |  |  |  |  |  |  |  |  |  |  |  |  |  | M9 |
|  |  |  |  |  |  |  |  |  |  |  |  |  |  |  |  | M12 |
| 47 | MY013  Age: 4+ yrs  Occupation: Teacher  Education Level: Bachelors  Economic Status: Middle income | F | + | + | + |  |  | + |  |  |  |  |  | + | + | Day 0 - 7 |
|  |  |  | + | + | + |  |  |  |  |  |  |  |  | + | + | M2 |
|  |  |  | + | + |  |  |  |  |  |  |  |  |  |  |  | M4 |
|  |  |  |  |  |  |  |  |  |  |  |  |  |  |  |  | M6 |
|  |  |  |  | + | + |  |  |  |  |  |  |  |  | + |  | M9 |
|  |  |  |  | + | + |  |  |  |  |  |  |  |  | + |  | M12 |
| 48 | MY014  Age: 59 yrs  Occupation: Banker/service holder  Education Level: Bachelors  Economic Status: High income | F | + | + | + |  |  | + |  |  |  |  |  | + | + | Day 0 - 7 |
|  |  |  |  |  |  |  |  |  |  |  |  |  |  |  |  | M2 |
|  |  |  |  |  |  |  |  |  |  |  |  |  |  |  |  | M4 |
|  |  |  |  |  |  |  |  |  |  |  |  |  |  |  |  | M6 |
|  |  |  |  |  |  |  |  |  |  |  |  |  |  |  |  | M9 |
|  |  |  |  |  |  |  |  |  |  |  |  |  |  |  |  | M12 |

*‘+’ sign denotes presence*

N.B.: Economic status was assigned based on per capita income of the family of the patient. Of note, all the patients participated in the LCA, were residents of city corporation areas.

**S3 Table** **B***: Outcomes of McNemar test.*

| **Account** | ***p* value  (from McNemar Test, Binomial Distribution)** |
| --- | --- |
| M2 vs Day 0 - 7 | <0.01 |
| M4 vs M2 | <0.01 |
| M4 vs Day 0 - 7 | <0.01 |
| M6 vs M4 | <0.01 |
| M6 vs M2 | <0.01 |
| M6 vs Day 0 - 7 | <0.01 |
| M9 vs M6 | <0.05 |
| M9 vs M4 | <0.01 |
| M9 vs M2 | <0.01 |
| M9 vs Day 0 - 7 | <0.01 |
| M12 vs M9 | <0.05 |
| M12 vs M6 | <0.05 |
| M12 vs M4 | <0.01 |
| M12 vs M2 | <0.01 |
| M12 vs Day 0 - 7 | <0.01 |

**S3 Table** **C***: Number of patients visited a physician due to post-CHIKV complications (n = 48).*

| **Time-point** | **Respondents (n = 48)**  **n, %** | ***p* value** |
| --- | --- | --- |
| M2 | 0, 0 |  |
| M4 | 0, 0 |  |
| M6 | 4, 8.33 | 0 .427 |
| M9 | 12, 25 | 0.0003 |
| M12 | 16, 33.33 | < 0.01 |
